# The Tsimane Health and Life History Project: Integrating anthropology and biomedicine

**DOI:** 10.1002/evan.21515

**Published:** 2017-04-21

**Authors:** Michael Gurven, Jonathan Stieglitz, Benjamin Trumble, Aaron D. Blackwell, Bret Beheim, Helen Davis, Paul Hooper, Hillard Kaplan

**Affiliations:** ^1^ Department of AnthropologyUniversity of California‐Santa BarbaraSanta Barbara CA; ^2^ Institute for Advanced Study in ToulouseToulouseFrance; ^3^ Center for Evolution and Medicine; School of Human Evolution and Social ChangeArizona State UniversityTempeAZ; ^4^ Department of Human Behavior, Ecology and CultureMax Planck Institute for Evolutionary AnthropologyLeipzigGermany; ^5^ Department of AnthropologyUniversity of UtahSalt Lake CityUT; ^6^ Santa Fe InstituteSanta Fe NM; ^7^ Department of AnthropologyUniversity of New MexicoAlbuquerqueNM

**Keywords:** evolutionary anthropology, behavioral ecology, evolutionary medicine, aging, cooperation

## Abstract

The Tsimane Health and Life History Project, an integrated bio‐behavioral study of the human life course, is designed to test competing hypotheses of human life‐history evolution. One aim is to understand the bidirectional connections between life history and social behavior in a high‐fertility, kin‐based context lacking amenities of modern urban life (e.g. sanitation, banks, electricity). Another aim is to understand how a high pathogen burden influences health and well‐being during development and adulthood. A third aim addresses how modernization shapes human life histories and sociality. Here we outline the project's goals, history, and main findings since its inception in 2002. We reflect on the implications of current findings and highlight the need for more coordinated ethnographic and biomedical study of contemporary nonindustrial populations to address broad questions that can situate evolutionary anthropology in a key position within the social and life sciences.

## 1 Introduction

Pioneering research among the Ju/'hoansi (Dobe! Kung) of Botswana and Namibia (Howell, 1979, 2010; Lee & DeVore, 1976), the Ache of Paraguay (Hill & Hurtado, 1996) and Hadza of Tanzania (Blurton Jones, 2016; Marlowe, 2010) helped pave the path toward long‐term prospective study of the demography, socio‐ecology and life history of contemporary foraging populations. These and other vital empirical studies of nonindustrial populations identified universal features of the evolved human life history: an encephalized brain, extended postreproductive life span, delayed juvenile growth, high fertility with multiple dependents, extensive intra‐ and intergenerational resource transfers and cumulative culture (Hill, Barton, & Hurtado, 2009; Kaplan, Hill, Lancaster, & Hurtado, 2000; Kaplan, 1997). This adaptive complex of traits helped humans colonize nearly all of earth's terrestrial and coastal ecosystems, with rapid demographic and geographic expansion beginning ∼45,000‐60,000 years ago (Henn, Cavalli‐Sforza, & Feldman, 2012). Although the exact timing and context of the evolution of these traits remain difficult to ascertain, a valuable approach for learning about this human adaptive complex is the holistic study of contemporary nonindustrial populations with limited access to modern amenities such as sanitation, health care, and labor‐saving technology.

In designing the Tsimane Health and Life History Project (THLHP), our goal was to integrate traditional ethnography with advances in methods and concepts from demography, economics, psychology, epidemiology, gerontology, and biomedicine. To test competing models of human life history evolution (Hawkes, 2003; Kaplan et al., 2000), we seek to relate changes in economic productivity, resource transfers, and social networks to changes in physical growth, development, and aging. A classic expectation from life‐history theory is that low exogenous mortality is a prime driver shaping a slower life history — that is, one involving prolonged maturation, greater investments in maintenance, and longer life (Stearns, 1992). A more sophisticated approach treats mortality as endogenous and co‐evolving with other life history traits, such as the role of learning in development (Kaplan & Robson, 2002). The learning‐intensive foraging niche shifted an already slow life history further in this direction, but questions remain: How did increased investments in learning and mortality reduction co‐evolve and how are they related to mortality‐reducing effects of human sociality, risk buffering, and cumulative culture? How did this ecological shift affect food production and sharing, risk reduction, and mating patterns? How do different sources of morbidity, such as exposure to a diverse array of pathogens and co‐morbidities, affect growth rates, maintenance costs, and aging? To what extent are human‐specific traits a coordinated and coevolved bundle versus a mix of adaptations and byproducts? To address these and other questions, we need to better understand: causes of morbidity and mortality in the absence of health care and other modern amenities; how different organ systems function by age in nonindustrial settings; and the social structure that may have been both a cause and effect of reducing morbidity and mortality. Joint behavioral and biomedical inquiry among contemporary nonindustrial societies like the Tsimane thus forms a core element of reconstructing patterns of human aging, health, life history, and sociality among ancestral humans.

We also aim to study how “modernization,” defined here as a trend toward greater participation in the market or cash economy, affects health and reshapes aspects of social ecology. Contemporary nonindustrial populations like the Tsimane are experiencing rapid social, economic, and cultural change. Socioeconomic transformation caused by increasing access to cash economies, wage labor, schooling, sanitation, and access to both modern medicine and amenities adds layers of complexity to defining how changing conditions alter health, risk management, and life history. Careful study is necessary for evaluating whether chronic diseases, such as cardiovascular disease (CVD) and diabetes, were common during human evolutionary history or are novel, resulting from an “evolutionary mismatch” as a consequence of the faster pace of lifestyle and environmental change as compared to genetic change.

These guiding research questions and themes about the human life course motivated inception of the THLHP. Over the last two decades, new research questions and directions grounded in life‐history theory have arisen, building on existing findings and contributing to important debates across multiple disciplines.

**Figure 1:**
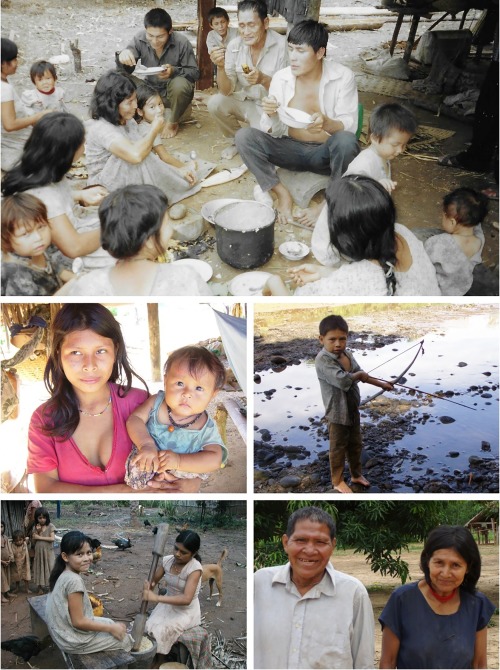
The Tsimane. Extensive sociality, a central feature enabling delayed childhood, high fertility, and long life span, is manifest in cooperative production, distribution, and child care. Photo credits: Michael Gurven

## 2 Beyond hunter‐gatherers

Hunter‐gatherers would be ideal case study populations, given that humans lived as hunter‐gatherers for most of our evolutionary history. However, most extant foraging populations are too small to adequately test causal models, especially among older adults. A broader range of societies is also required to understand evolved human reaction norms across different environments during recent millennia. Subsistence horticulturalists who also hunt, gather, and fish share many similarities with existing full‐time hunter‐gatherers, including natural fertility, minimal access to modern sanitation or medicine, and limited group size. Differences between foragers and horticulturalists can shed light on the impacts of plant and animal domestication on health and life‐history‐relevant traits, including parasite burden, nutritional status, fertility, mobility, residence patterns, and social structure. Bottlenecks and expansions of human populations during the advent of agriculture also had profound effects on human population genetics (Fumagalli, Sironi, Pozzoli, Ferrer‐Admettla, Pattini, & Nielsen, 2011; Hawks, Wang, Cochran, Harpending, & Moyzis, 2007), further highlighting the importance of nonforagers when considering health and disease in contemporary populations.

## 3 The tsimane of bolivia

**Figure 2:**
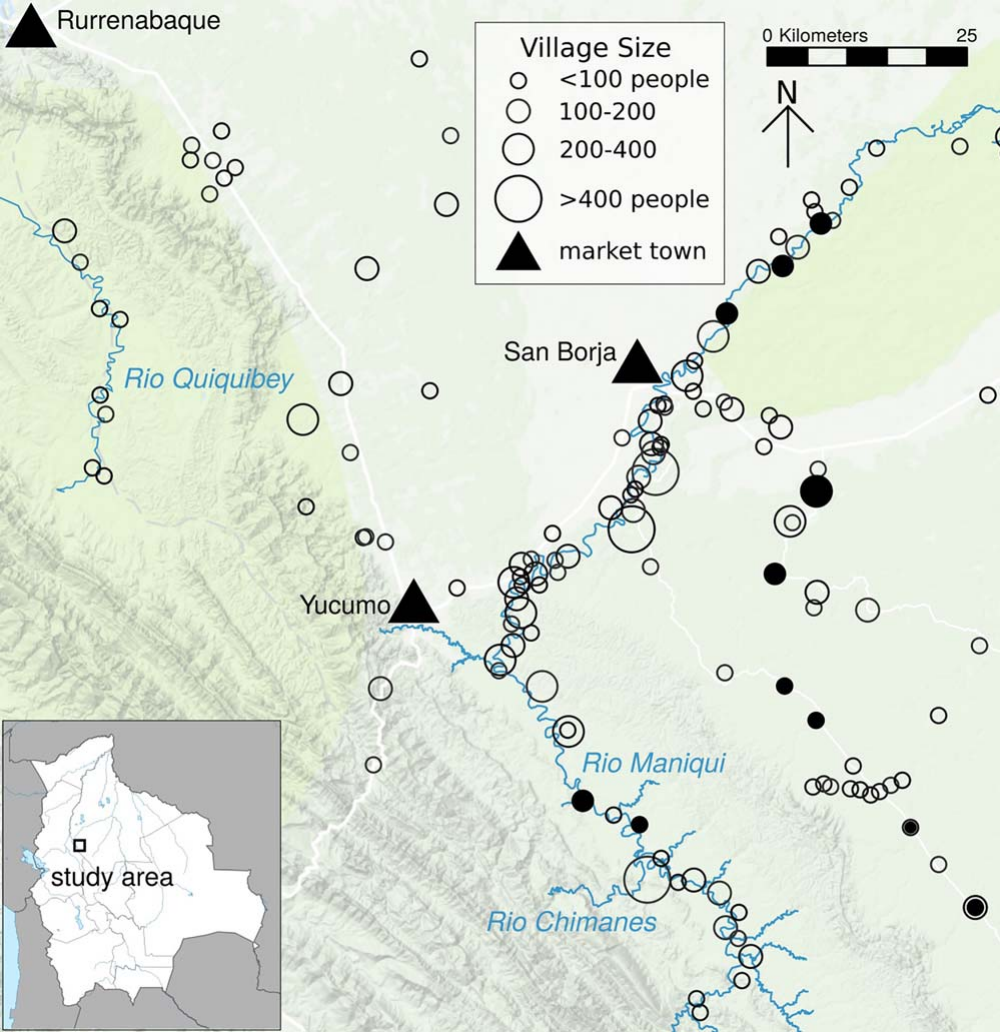
Map of Tsimane territory and study villages. Solid circles signify “core” villages where relatively long‐term study has occurred; empty circles are other villages visited by the biomedical team; triangles reflect towns. Sizes of circles are proportional to village census size

Bolivia is home to 36 indigenous groups that together constitute over 60% of the population (INE, 2012). Of the 30 groups inhabiting the tropical lowlands, the Tsimane are among the most isolated (along with the Yuqui and Siriono) (Figure 1). The Tsimane are forager‐horticulturalists of the Bolivian Amazon who subsist on slash‐and‐burn horticulture (mainly plantains, rice, sweet manioc, and corn); fishing in rivers, streams and lagoons; hunting a large array of neotropical mammals; and seasonal gathering of fruits and other foods, such as honey and nuts. They inhabit more than 90 villages numbering from 50 to 500 individuals along the Maniqui, Quiquibey, and Mato Rivers and interfluvial *terra firme* (Figure 2). While early censuses in the late 1990s estimated a population size of 6,000 Tsimane, the most recent THLHP census in 2015 suggested a number closer to 16,000 (also see INE (2012)) and a population growth rate of over 3.5%. Unlike most extant foragers, the relatively large Tsimane population provides the opportunity for study of all stages of the human life course, including late adulthood, which is essential to study competing models of human life‐history evolution.

Throughout the first half of the twentieth century, Tsimane maintained a traditional lifestyle because of the relative absence of navigable roads in their territory. Road and river access has been improved by logging, development projects and new technologies such as the recent boom of *pequi* outboard boat motors in the last five years, but access remains somewhat limited for many villages, especially during certain periods of the year when heavy rains wash out bridges and dirt roads and make river travel dangerous. This variable access to the market and associated nontraditional cultural influences act as a quasi‐experimental opportunity for examining effects of socioeconomic change on decision‐making (McAllister, Gurven, Kaplan, & Stieglitz, 2012), economic production (Schniter, Gurven, Kaplan, Wilcox, & Hooper, 2015), social networks (Rucas, Gurven, Winking, & Kaplan, 2012), sharing and risk management strategies (Gurven, 2004b; Gurven, Jaeggi, von Rueden, Hooper, & Kaplan, 2015), and health (Gurven, 2012b; Gurven, Blackwell, Rodríguez, Stieglitz, & Kaplan, 2012; Gurven, Jaeggi, Kaplan, & Cummings, 2013; Trumble, Stieglitz, Rodriguez, Linares, Kaplan, & Gurven, 2015). Many of these outcomes also affect the pace of socioeconomic change, thus providing additional opportunities to examine the relative importance of the determinants underlying market participation.

## 4 Research design and sample

**Figure 3:**
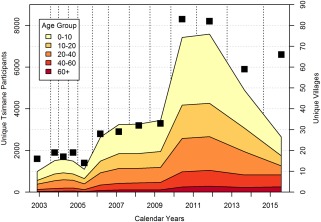
Age structure of biomedical surveillance by calendar year (2002‐2015). Colored polygons show the number of unique individuals sampled in each age group, while black boxes indicate the number of unique villages sampled. Over the sample period, there were 13 rounds (indicated by vertical dotted lines) of varying length (mean ± SD = 0.99 ± 0.40 yrs, range 0.42‐1.80 yrs). In 2013‐2015, THLHP sampled all adults age 40+ each round, but partitioned the <40 age group sampling into two or three rounds [Color figure can be viewed at wileyonlinelibrary.com]

**Figure 4:**
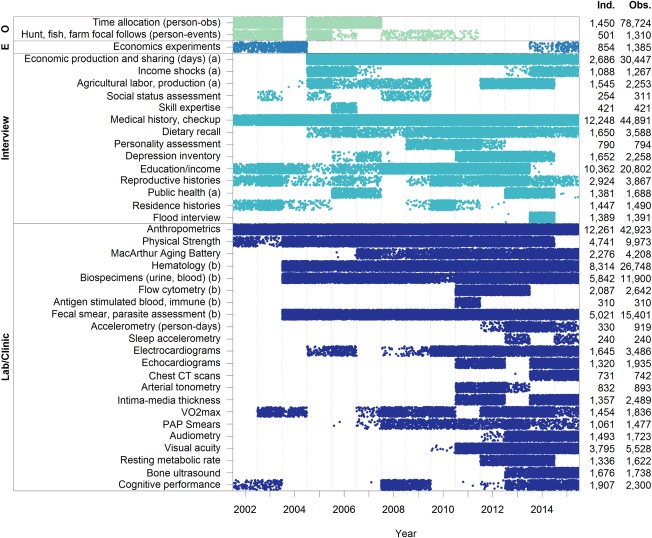
Summary of data types and sample sizes. Color of bars refers to data type. From top to bottom: behavioral observations (O), experiments (E), interview, laboratory/clinical. Data points are distributed along the x‐axis according to the year of their collection ^a^ reflects household‐level data; otherwise data are at the individual level. ^b^The following measures are obtained from biospecimens: FECES ‐ presence or absence of 17 parasites, helminth egg burden; SALIVA, URINE ‐ reproductive hormones; URINE – C‐peptide, oxidative stress; BLOOD – Leukocyte count and subsets, cytokines, immunoglobulins, ESR, DNA, blood lipids [Color figure can be viewed at wileyonlinelibrary.com]

The THLHP, codirected by HK and MG, officially began in 2002 after initial excursions by MG to Tsimane territory in 1999. From its inception, the research design has included a mobile biomedical team composed of Bolivian physicians, biochemists, and Tsimane research assistants. This team visits each village roughly once a year to provide broad snapshots of physical condition and health, demography, and socioeconomic life (Figure 3). A reduced “core team” of a few anthropologists and Tsimane research assistants complements this mobile team through more focused, longer‐term sampling and more intensive economic data collection in core villages (Figure 4). These extended field sessions in core villages provide rich ethnographic study of economic and social behavior and health.

Funding was initially provided by the National Science Foundation in 2001 and 2004, and then, beginning in 2004, continuously by the National Institutes of Health/National Institute on Aging. NIH/NIA support permitted expansion of project scope and content. Thus, not only sampling, but the breadth and depth of data collection have expanded considerably over the project's duration (Figures 3 and 4). Our initial sample included 18 villages, which then expanded to 23 by 2005, 85 by 2009, and 90 by 2015. In addition to employing Bolivian biomedical and administrative project personnel, the THLHP has collaborated with an international and interdisciplinary team of cardiologists, gerontologists, demographers, economists, biologists, psychologists, and epidemiologists (Box 1). To date, 17 Ph.D. dissertations and 8 undergraduate honors theses have been written in collaboration with the THLHP. Numerous graduate students and postdoctoral scholars have assisted in many aspects of project operations (see www.unm.edu/~tsimane).

Our holistic bio‐behavioral approach has the advantage that we can link information on multiple phenotypes for the same individual over time to better understand factors influencing aging, health, and sociality. Our team has collected systematic baseline data at the individual level on many phenotypic traits: demographic, behavioral, morbidity, and biomarkers related to health and aging, infectious exposure, inflammation, and other indicators of immune function, as well as measures of physical and functional status (Figure 4). The span of measures is derived from surveys, physical exams, and biospecimens (blood, feces, urine, saliva). Several THLHP protocols were modified from prior life‐course epidemiological studies in high income countries (e.g., NHANES), permitting direct cross‐cultural comparisons (Box 1).

Since 2007, the THLHP has focused on aging, particularly the examination of associations among atherosclerosis, cardiovascular health, and immune function. While Tsimane experience high levels of inflammation, which is implicated in nearly all stages of atherosclerosis and is a strong risk factor for heart disease (Gurven, Kaplan, Winking, Eid, Vasunilashorn, Kim, Finch, & Crimmins, 2009), Tsimane also engage in demanding physical activity throughout life, show minimal obesity, and have limited food intake, which are protective against heart disease. Given the opposing effects that these factors may have on chronic disease risk, understanding the impact of inflammation in an infectious context is an important goal. To this end, we have started investigating relationships between cardiovascular and metabolic outcomes, infectious load, inflammatory and anti‐inflammatory immune activation, genetic susceptibility variants, functional status, VO_2_max, physical activity and diet, economic productivity and social roles, and psychosocial factors. Collection of diverse bio‐behavioral and demographic data enables a comprehensive analysis of the effects of modernization and social processes on these health outcomes. Thus, our research program attempts to advance a “whole organism” understanding of the aging process in environmental conditions similar to those in which humans evolved, while also studying reaction norms and potential health‐related mismatches as those environmental conditions change.

## 5 What have we learned so far?

New THLHP findings replicate those from earlier studies of foragers and forager‐horticulturalists, demonstrating a skill‐intensive economic niche with a long learning period, biparental investment, socially mediated risk‐buffering, multigenerational resource transfers by parents and grandparents to dependent young, high productivity of postreproductive adults until the eighth decade of life, and a long adult life span. In fact, none of the patterns found in early studies have been contradicted by Tsimane research. However, this new research has enriched our understanding of: (1) life span and the aging process; (2) the role of infectious disease in shaping different life history components, including growth and senescence; (3) chronic disease and lifestyle change; (4) the life history of production, consumption and cooperation; (5) marriage, parental investment, and conflicts of interest between spouses; (6) status striving, personality, and determinants of reproductive success; and (7) how modernization affects traditional lifeways (see Table 1). In what follows, we discuss each theme in turn.

**Table 1:** Research Domains and Foci of the THLHP. To date, 109 peer‐reviewed publications in 42 scientific journals or as book chapters spanning 14 disciplines have used THLHP data. In addition to anthropology, these publications have addressed important areas in gerontology, genetics, demography, physiology, epidemiology, economics, psychology, general medicine, cardiology, immunology, osteology, and biology.

DOMAIN	FOCI	RELEVANT CITATIONS
Aging, maintenance and mortality	Immune function	Blackwell et al., 2011; Blackwell et al., 2013; Blackwell et al., 2015; Blackwell et al., 2016; Gurven et al., 2016; Vasunilashorn et al., 2011
	Hormone‐behavior interactions	Jaeggi, Trumble, Kaplan, & Gurven, 2015; Trumble et al., 2012; Trumble et al., 2013; Trumble, Smith, O'Connor, Kaplan, & Gurven, 2014; von Rueden, Trumble, et al., 2014
	Physical senescence	Kaplan et al., 2010; Pisor et al., 2013; Trumble, et al., 2015
	Infection	Blackwell et al., 2011; Blackwell et al., 2013; Blackwell et al., 2015; Gurven, et al., 2008; Gurven, et al., 2009; Gurven, Trumble, Stieglitz, Rodriguez, Linares, Kaplan, & Gurven, 2016; Martin, Blackwell, Gurven, & Kaplan, 2013; Stieglitz, Blackwell, et al., 2012; Stieglitz, et al., 2015; Vasunilashorn et al., 2010; von Rueden, Trumble, et al., 2014
	Cardiovascular disease	Gurven, Blackwell, et al., 2012; Gurven, Kaplan, et al., 2009; Gurven, Trumble, Stieglitz, et al., 2016a; Horvath et al., 2016; Vasunilashorn et al., 2010
	Genetics	Horvath et al., 2016; Vasunilashorn et al., 2011
	Life history theory	Blackwell et al., 2015; Gurven, 2012a; Gurven, Costa, et al., 2016; Gurven & Kaplan, 2008; Gurven et al., 2006; Gurven, Stieglitz, et al., 2012; Hooper, Gurven, & Kaplan, 2014; Hooper, Gurven, et al., 2015; Kaplan, Gurven, & Winking, 2009; Kaplan et al., 2010; Kaplan et al., 2015; McAllister et al., 2012; Schniter et al., 2015; Walker et al., 2006; Walker, Gurven, Burger, & Hamilton, 2008
	Bone strength	Stieglitz, Beheim, et al., 2015; Stieglitz, Madimenos, Kaplan, & Gurven, 2016
	Bio‐demography	Gurven, 2012b; Gurven & Fenelon, 2009; Gurven & Kaplan, 2007; Gurven & Kaplan, 2008; Gurven et al., 2007; Hooper et al., 2014; Kaplan & Gurven, 2008; Kaplan, Gurven, et al., 2009; Kaplan et al., 2010; Tuljapurkar, Puleston, & Gurven, 2007
	Psychological well‐being	Stieglitz, Jaeggi, et al., 2014; Stieglitz, Schniter, et al., 2014; Stieglitz, Trumble, et al., 2015
	Cognition	Gurven, Fuerstenberg, et al., 2016; Trumble, Gaulin, Dunbar, Kaplan, & Gurven, 2016; Trumble, Stieglitz, Thompson, et al., 2015
Physical activity and performance	Sleep	Yetish et al., 2015
	Metabolism	Gurven, Trumble, Stieglitz, Yetish, et al., 2016
	Functional ability	Pisor et al., 2013; Stieglitz, Jaeggi, et al., 2014; Stieglitz, Schniter, et al., 2014
	Mobility and migration	Gurven, Jaeggi, et al., 2013; Jaeggi et al., 2015; Miner, Gurven, Kaplan, & Gaulin, 2014; Trumble et al., 2014
Cooperation and fairness	Social preferences	Gurven, 2004a; Gurven, 2004b, 2012a; Gurven, Stieglitz, et al., 2012; Gurven & Winking, 2008; Gurven, Zanolini, & Schniter, 2008; Gurven, 2014; Henrich et al., 2005; Henrich et al., 2010; Henrich et al., 2006; Kaplan, Hooper, & Gurven, 2009; Marlowe et al., 2008; Marlowe et al., 2010; Straub et al., 2010; Walker et al., 2013
	Food sharing	Gurven & Von Rueden, 2006; Hooper et al., 2014; Hooper, DeDeo, Caldwell Hooper, Gurven, & Kaplan, 2013; Hooper, Gurven, et al., 2015; Jaeggi & Gurven, 2013; Jaeggi et al., 2016; Stieglitz, et al., 2016
	Social networks	Gurven et al., 2015; Hooper et al., 2014; Hooper et al., 2013; Hooper, Demps, Gurven, Gerkey, & Kaplan, 2015; Hooper, Gurven, et al., 2015; Jaeggi et al., 2016; Pisor & Gurven, 2016; Rucas et al., 2006a; Rucas et al., 2012; Rucas, Gurven, Kaplan, & Winking, 2010; Stieglitz, Schniter, Von Rueden, Kaplan, & Gurven, 2014
	Inequality	Borgerhoff Mulder et al., 2009; Gurven et al., 2015; Gurven et al., 2010; Stieglitz, Gurven, Kaplan, & Hooper, 2016; von Rueden, et al., 2014
	Leadership	Glowacki & von Rueden, 2015; von Rueden, Gurven, Kaplan, & Stieglitz, 2014
	Morality	Barrett et al., 2016; Fessler et al., 2015; Gurven, 2014; Henrich et al., 2010; Henrich et al., 2006; Marlowe et al., 2008
Conflict and aggression	Intrasexual competition	Rucas et al., 2006a; Rucas et al., 2012; Rucas et al., 2010; Trumble et al., 2012; Trumble et al., 2013
	Domestic violence	Stieglitz, et al., 2012; Stieglitz et al., 2011
	Formidability	Sell et al., 2010; Sell et al., 2009
Ontogeny	Somatic growth	Blackwell et al., in press; Stieglitz, et al., 2015; Veile, Winking, Gurven, Greaves, & Kramer, 2012; Walker et al., 2006
	Task delegation	Stieglitz, Gurven, Kaplan, & Hooper, 2013
	Skills acquisition and learning	Gurven et al., 2016; Kaplan, Gurven, & Winking, 2009; Kaplan et al., 2010; Schniter et al., 2015
	Intergenerational transmission	Borgerhoff Mulder et al., 2009; Gurven et al., 2010; Gurven, et al., 2012; Schniter et al., 2015
	Breastfeeding or complementary feeding	Han et al., 2016; Martin et al., 2012; Veile et al., 2014
	Puberty and adolescence	Hodges‐Simeon, Gurven, Cárdenas, & Gaulin, 2013; Hodges‐Simeon, Gurven, & Gaulin, 2015; Hodges‐Simeon, Gurven, Puts, & Gaulin, 2014; Hodges‐Simeon, Sobraske, Samore, Gurven, & Gaulin, 2016
	Personality	Gurven, von Rueden, Massenkoff, Kaplan, & Lero Vie, 2013; Gurven et al., 2014; von Rueden et al., 2015
Food production and livelihood	Foraging	Gurven, 2012a; Gurven et al., 2006; Hooper, Demps, Gurven, M., Gerkey, D., & Kaplan, 2015; Jaeggi et al., 2015; Schniter et al., 2015; Trumble et al., 2014
	Horticulture	Gurven et al., 2010; Trumble et al., 2013
	Division of labor	Gurven, 2012a; Gurven, et al., 2009; Hooper, et al., 2015; Jaeggi et al., 2016; Kaplan, Hooper, & Gurven, 2009; Stieglitz et al., 2013
	Social status	Glowacki & von Rueden, 2015; Gurven & Von Rueden, 2006; Von Rueden, Gurven, & Kaplan, 2008; von Rueden et al., 2011; von Rueden, Gurven, et al., 2014; von Rueden, et al., 2014
Mating and reproduction	Mate acquisition	Stieglitz, Blackwell, et al., 2012; Winking et al., 2013
	Marriage	Gurven, 2012a; Gurven, et al., 2009; Stieglitz, et al., 2012; Stieglitz, et al., 2016; Stieglitz, et al., 2012; Stieglitz et al., 2011; Winking, 2006; Winking, Gurven, Kaplan, & Stieglitz, 2009; Winking et al., 2007; Winking et al., 2013
	Fertility	Blackwell et al., 2015; Gurven et al., 2014; Kaplan et al., 2010; Tuljapurkar et al., 2007; von Rueden et al., 2011; Winking et al., 2013
	Parental investment	Gurven, et al., 2009; Hooper et al., 2014; Hooper, et al., 2015; Hooper, et al., 2015; Winking, 2006; Winking & Gurven, 2011; Winking, Gurven, & Kaplan, 2011a, 2011b; Winking et al., 2009; Winking et al., 2007
	Fertility transition	Kaplan et al., 2015; McAllister et al., 2012
	Costs of reproduction	Gurven, et al., 2016; Stieglitz, et al., 2015

### 5.1 Life span, aging and well‐being

From the period 1950‐1989, life expectancy at birth among Tsimane was 43 years; by 2002, life expectancy increased to about 53 years (Gurven, Kaplan, & Zelada Supa, 2007). Despite recent improvement, Tsimane death rates at all ages are similar to those in Europe in the 1800s (Gurven, Kaplan, Crimmins, Finch, & Winking, 2008). Unlike the typical pattern observed historically, in which initial increases in life span are largely caused by reductions in infant and child mortality, the improvement from 1990‐2002 was more a result of reduced death rates in adulthood than during infancy or childhood. We suspect that this is due to differences in access to medical interventions for adults and older children, since they have a greater ability than do infants or young children to seek and survive treatment. Despite recent improvement in access to health care facilities, Tsimane cultural beliefs about sickness and death, coupled with some ethnic discrimination in town, may still deter people from seeking treatment. The modal age of adult death is 70 years (SD = 6.3), similar to that among hunter‐gatherers and other horticulturalists and 1.5 decades earlier than that in high‐income countries (Gurven & Kaplan, 2007).

Box 1 **THLHP in Comparative Context**

The THLHP uses cross‐sectional and longitudinal biomedical, epidemiological, demographic, and anthropological methodologies that are directly comparable to methods used in the U.S., other developed countries, and the developing world (Figure 4). Those comparisons illuminate the evolved aging process manifested in demographic, epidemiological, functional, and behavioral outcomes, as well as how changes in current environments interact with this evolved system to produce modern outcomes. Although the THLHP involves comprehensive study of many topics in a single population (Table 1), a broader perspective requires comparative study with a wider range of variation in key predictors. To this end, Tsimane data have been applied to comparative studies of mortality (Gurven & Kaplan, 2007), fairness (Henrich et al., 2005; Henrich et al., 2006), morality (Barrett et al., 2016; Fessler et al., 2015), wealth inheritance and inequality (Borgerhoff Mulder et al., 2009), postmarital residence (Walker et al., 2013), physical growth rates (Walker et al., 2006), sleep (Yetish et al., 2015), and sexual division of labor (Stieglitz, Gurven, Kaplan, & Hopfensitz, 2016). Ongoing comparative studies involving Tsimane include cardiovascular disease, bone strength, diabetes, epigenetics and aging, personality structure, breast milk composition, cooperation, and jealousy. The THLHP has also influenced the design of other projects, such as the Shuar Health and Life History Project, and recent studies among Agta and Pygmies.

**Figure 5:**
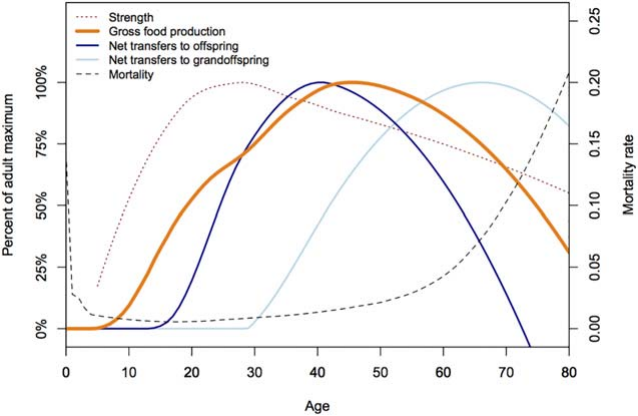
Age profiles of caloric production, net food transfers to children and grandchildren, physical strength, and mortality. Though physical growth and strength peak in early adulthood, food production ability peaks in the fifth decade of life. Transfers to children peak by around age 40, while those to grandchildren peak in the 60s. Horticulture increases the productivity of older adults more than would be expected among hunter‐gatherers. By the age of 70 years, productivity and transfers decline while mortality increases rapidly [Color figure can be viewed at wileyonlinelibrary.com]

By the age of 60 years, Tsimane show evidence of significant physical disability. Physical strength declines continuously by the fourth decade of life (Gurven, Kaplan, & Gutierrez, 2006) (Figure 5). At the age of 60 years, more than 60% of Tsimane complain about hearing loss, more than 80% have trouble seeing close distances, and more than 70% can no longer chop large trees in their fields. About 50% of men and 70% of women over age 70 can no longer walk long distances and frequently complain about painful arthritis in their legs, back, and hips. Over 70% of men no longer hunt by age 70; these men complain about weakness, lethargy, and having poor eyesight and hearing. Functional disability is a strong predictor of Tsimane depression: adults aged 50+ years in the top decile of a composite disability measure score 14% higher on a depression scale than those in the bottom decile after controlling for multiple potential confounders (Stieglitz, Schniter, Von Rueden, Kaplan, & Gurven, 2014). Depression increases with age, as disability increases and limits production and sharing ability, which runs counter to the common claim that human depression universally peaks in mid‐adulthood (i.e., mid‐life crisis) (Weiss, King, Inoue‐Murayama, Matsuzawa, & Oswald, 2012).

Alongside late life declines in strength, coordination, and functional status, several components of adaptive immune function show evidence of rapid senescence. Naïve CD4+ helper T‐cells, essential for mobilizing immune defenses against unfamiliar pathogens, are considerably depleted by age 50, while natural killer cell counts are substantially elevated (Blackwell, Trumble, Maldonado Suarez, Stieglitz, Beheim, Snodgrass, Kaplan, & Gurven, 2016). Consistent with these patterns, a measure of epigenetic age acceleration, based in part on the estimation of immune cell counts, is higher among Tsimane than among other populations (Horvath, Gurven, Levine, Trumble, Kaplan, Allayee, Ritz, Chen, Lu, Rickbaugh, Jamieson, Sun, Li, Chen, Quintana‐Murci, Fagny, Kobor, Tsao, Reiner, Edlefsen, Absher, & Assimes, 2016).

Box 2 **Research Ethics and Community‐Based Participatory Research**

Given the high morbidity, mortality, and lack of public health infrastructure in the Tsimane region, our code of ethics for conducting long‐term fieldwork in Bolivia is to provide aid and assistance where possible, from specialized medical care on a case‐by‐case basis to humanitarian efforts following environmental disasters. To facilitate these efforts, formal agreements have been established and maintained with several Bolivian institutions, including universities; medical schools; national, regional and local governments; hospitals; national laboratories; and nongovernmental aid institutions. In conjunction with epidemiological data collection efforts of the mobile biomedical team, project physicians treat Tsimane in their villages for various bacterial and fungal infections, abscesses, minor trauma, diarrhea, parasites, and other primary care needs. Since the project's inception, over 45,000 person‐visits with a physician providing clinical care have been conducted, with an average of 3,400 individual patient‐visits per study year. Core team members residing in villages for longer periods than the mobile team offer further primary care as needed by villagers. For Tsimane with more urgent needs requiring specialized medical care, we have facilitated their transport and treatment in San Borja or, when necessary, in Trinidad, Cochabamba, San Ignacio, or La Paz, while ensuring their proper recovery and follow‐up treatment if necessary. Since 2007, 456 Tsimane have received such specialized care. Examples of cases include hernia surgeries; osteomyelitis and fractured bones; cystocele, ovarian and abdominal tumors; congenital diseases; head trauma; brain tumors; severe burns; cataract and pterigion surgeries; and treatment for severe trauma (e.g., snakebite, machete lacerations). Aside from providing or facilitating medical care, the THLHP has also trained over 45 Tsimane as project personnel, many of whom have contributed critical advice on identifying problems, managing logistics, and navigating politics. The THLHP also has provided educational workshops on different aspects of disease detection and prevention and donated shortwave two‐way radios for intervillage and village‐to‐town communication. THLHP collaborators have also donated medical equipment to local hospitals (e.g., surgical supplies, endoscopes, EKG machine) and provided further clinical training to Bolivian medical professionals. Biannual reports (in Spanish) including results of Tsimane epidemiological surveillance are provided to the Tsimane political council (*Gran Consejo Tsimane*) and local authorities to help solicit government resources to meet the health care needs of the Tsimane.

Box 3 **Strengths and Limitations**

Longitudinal studies are the gold standard for studying life‐course processes (e.g., development, aging) and linkages across life stages, but they are rare in anthropology. The ability to prospectively link behavioral, psychological, health, and other data from the same individuals is a major strength of the THLHP design. Yet the broad range of topics covered by THLHP is both its main strength and weakness. Given limited personnel, some topics are given more attention and studied in greater depth than others. The duration of longitudinal data varies by data type and longitudinal data do not exist for some protocols. However, most data, even if preliminary, have helped motivate further study with existing and new collaborators. For example, initial data on age profiles of cognitive performance using a simple battery of psychometric tests has led to more focused studies of cognitive aging and dementia.

For biomedical surveillance, coverage and acceptance rates are very high (>85%), helping to ensure representativeness and minimize selection bias. High participation rates are possible because we have developed long‐term trusting relationships in the villages and close ties with relevant institutions such as the *Gran Consejo Tsimane* and other local political groups. Providing medical care and training Tsimane in health promotion has greatly improved our reception in the area, helping avoid loss to follow‐up and contributing to Tsimane human capital development. Another strength has been to develop the local Bolivian infrastructure to permit field‐friendly methodologies comparable to those used in more traditional laboratory or clinical settings, including echocardiograms, ultrasounds, CT scans, flow cytometry (Blackwell et al., 2016), tissue doppler, and in‐vitro antigen stimulation (Stieglitz, Trumble, et al., 2015; Trumble, Blackwell, et al., 2016). This capacity building and advanced training has been an important part of our continued success in working with local hospitals and other institutions.

In late life, fluid cognitive abilities related to reasoning and processing speed also appear to decline from their peak in early adulthood, whereas crystallized abilities based on cumulative experience and knowledge increase throughout the life span (Gurven, Fuerstenberg, Trumble, Stieglitz, Beheim, Davis, & Kaplan, 2016; Trumble, Stieglitz, Thompson, Fuerstenberg, Kaplan, & Gurven, 2015). While this pattern has been widely documented in Western contexts, it had never before been systematically assessed in a nonliterate or nonindustrial population. While the decline in fluid abilities seems to mirror changes in physical abilities with age, nondeclining crystallized abilities are consistent with the functional role of middle‐aged and older adults as mentors, instructors, and caregivers in Tsimane society. Resource production is positively associated with psychological well‐being for older adults (Stieglitz, et al., 2014), which is consistent with a human life‐history perspective emphasizing the importance of adult economic production surplus and downward net transfers.

In summary, mortality rate increases in late adulthood are linked to changes in phenotypic condition due to aging and associated declines in muscularity and physical strength, aerobic fitness, sensory acuity, and immune function. Those changes, in turn, are linked to changes in economic productivity and psychological well‐being. The productivity of Tsimane adults supports net economic transfers to descendants until the eighth decade, coinciding with the modal age of adult death among Tsimane and other subsistence populations (Gurven & Kaplan, 2007). These findings demonstrating declining fitness‐related utility at late ages are compatible with the “disposable soma” theory of aging, which views aging as the result of compromised energy allocation favoring investments providing fitness benefits earlier in life (e.g., reproduction) in light of somatic maintenance costs that increase with age. However, despite speculation, it still has not been clearly determined why humans live as long as they do but not longer. Our working hypothesis is that the fitness benefits of costly investments in grandchildren outweigh the fitness costs of slowing down the aging process, but that these benefits diminish once grandchildren are past the high‐mortality period early in life. Given dispersal, migration, and the dilution of genetic relatedness with each successive generation, the sum of fitness effects over all descendants may be too small to favor further delays in aging.

### 5.2 Pathogens and life history

Tsimane exhibit high rates of diverse infections. Over 66% of Tsimane have at least one intestinal parasite, the most common being hookworm (*Ancylostoma duodenale* or *Necator americanus*, prevalence 56%), roundworm (*Ascaris lumbricoides*, 15%) and whipworm (*Trichuris* sp., 4%) (Blackwell, Gurven, Sugiyama, Matemeros, Lieber, Martin, Kaplan, & Snodgrass, 2011). Protozoan infections are also common, including *Giardia lambia* (30%) and *E. histolytica* (5%). About half of men and women have anemia, with children and adolescents showing the highest risk (56% for girls, 63% for boys). Polyparasitic co‐infection is not uncommon. Several helminth species co‐occur, whereas helminths such as hookworm and roundworm seem to have protective effects against giardia infection (Blackwell, Martin, Kaplan, & Gurven, 2013). Helminths also affect Tsimane fertility. Hookworm reduces fertility, while roundworm may shorten interbirth intervals, perhaps by increasing maternal immunological tolerance of a fetus (Blackwell Tamayo, Beheim, Trumble, Stieglitz, Hooper, Martin, Kaplan, & Gurven, 2015). Given the high transmission rate of multiple pathogens in the Tsimane environment, the prevalence of several parasites and investments in immune defenses against them (e.g. immunoglobulin‐E, IgE) peak earlier in childhood than in populations with lower transmission rates (Blackwell, Gurven, Sugiyama, Madimenos, Liebert, Martin, Kaplan, & Snodgrass et al., 2011). This finding is consistent with the “peak shift” hypothesis, which suggests that peak incidence rates occur at younger ages as immune defenses develop earlier in more infectious environments.

Infections are the main source of Tsimane morbidity and mortality over the life course (Gurven, Kaplan, & Zelada Supa, 2007). Gastrointestinal illness and respiratory infections are frequent: 30%‐40% of infants and young children suffer from each; 30%‐40% of adults suffer from gastrointestinal illness; and 20%‐30% of adults suffer from respiratory infections. Living in a pathogenic environment likely favors pro‐inflammatory (CRP, IL‐6) alleles (Vasunilashorn, Finch, Frimmins, Vikman, Stieglitz, Gurven, Kaplan, & Allaye, 2011) and higher levels of inflammation than do more hygienic environments. Levels of CRP, an indicator of inflammation, are higher than among Americans, especially in childhood (Blackwell et al., 2016). Cross‐sectional estimates of life lived with high CRP indicate that by the age of 34 years, Tsimane have spent an average of 15 years (42% of life) with high CRP whereas, in the U. S., the corresponding number is 6.8 years (19%). Tsimane CRP levels in early life are higher than those sampled among diverse populations, including Italians, Mexicans, Filipinos, and Native Americans (Gurven, et al., 2008). CRP levels vary between and within individuals, with half of the total variation being between individuals; thus, elevations likely do not represent only acute infections, which instead are moderately stable within individuals over time (Blackwell et al., 2016). Trichomoniasis is more prevalent among Tsimane living near town than among those living farther from town (Stieglitz, Blackwell, Gutierrez, Linares, Gurven, & Kaplan, 2012). In contrast, Tsimane living farther from town have higher CRP than those living near town, suggesting higher exposure to other infectious diseases in remote villages. Other biomarkers also suggest high levels of immune activity throughout life: Tsimane have higher levels of leukocytes, erythrocyte sedimentation rate, B cells, and natural killer cells than do Americans at all ages (Blackwell et al., 2016). Tsimane adults also show a higher rate of decline in naïve CD4 T helper cells than occurs in other populations, which might put them at greater health risk when exposed to new infections (Blackwell et al., 2016). On average, 20% of Tsimane white blood cells (WBCs) are eosinophils, consistent with high levels of parasitic infection, as compared with a U.S. reference range of <5%. Antibodies related to infection are also high among Tsimane: immunoglobulin‐G (IgG) levels are about twice as high and IgE, which is most relevant for helminthic infection, is about 100 times higher than typical U.S. levels (Blackwell et al., 2011).

Perhaps as a consequence of high levels of infection and immune activation, Tsimane have elevated resting metabolic rates and 10%‐15% of metabolism is associated with immune activation (Gurven, Trumble, Stieglitz, Yetish, Cummings, Blackwell, Beheim, Kaplan, & Pontzer, 2016). The high prevalence of infection and the shunting of requisite energy toward immune defense may help explain the slow somatic growth and stunting common among Tsimane, as well as similarly energy limited populations experiencing high pathogen burden (Blackwell, Urlacher, Beheim, von Rueden, Jaeggi, Stieglitz, Trumble, Gurven, & Kaplan, 2016). Population differences in growth trajectories during childhood may reflect patterns of pathogen exposure and immune investment, since Tsimane show slower growth during periods of their early peak IgE production (Blackwell et al., 2011). The higher energetic cost of tolerating and/or defending oneself from parasites may be further offset by other shifts in energy use. Possibilities in the Tsimane context include lower physical activity, sickness behavior (Stieglitz, et al., 2015), cachexia and osteopenia (Stieglitz, Beheim, Trumble, Madimenos, Kaplan, & Gurven, 2015), dyslipidemia and anemia (Gurven, Trumble, Stieglitz, Yetish, Cummings, Blackwell, Beheim, Kaplan, & Pontzer, 2016; Straub, Cutolo, Buttgereit, & Pongratz, 2010).

In summary, infectious disease has multiple phenotypic consequences. Not surprisingly, it appears to upregulate immune activity, resulting in greater energy expenditures throughout life and more rapid senescence of some immune cell populations. Variation in pathogen burden across human environments and over time within individuals seems to be associated with adaptive and plastic immune responses. The Tsimane, living in a warm, humid, tropical environment in relatively settled communities, may experience greater pathogen burden than other contemporary and ancestral human populations. Yet their modal age at death is similar to that of subsistence‐level populations living in drier environments (Gurven & Kaplan, 2007). Perhaps natural selection on human aging has resulted in a species‐typical life span despite differing sources of morbidity and death across populations.

### 5.3 Chronic disease, mismatch, and lifestyle change

The THLHP provides an opportunity to test ideas about the role of environmental and socioeconomic change on health concerns believed to be either universal aspects of human aging or consequences of an evolutionary mismatch between longstanding genetic adaptations and novel environments. We have found that several conditions common in urban areas of both high and low‐income countries are rare or absent among Tsimane. As noted elsewhere (Rook, 2012; Yazdanbakhsh, Kremsner, & van Ree, 2002), allergies, atopy, and other auto‐immune diseases are rare among Tsimane. This is to be expected, according to the “hygiene” and “old friends” hypotheses, which propose that early pathogenic exposures, especially to helminths, help promote improved immune regulation in ways that temper pro‐inflammatory conditions (Rook, 2012; Yazdanbakhsh, 2002). Benign prostate hyperplasia is also rare, presumably due in large part to lower testosterone levels in early adulthood than those in Western populations (Trumble, Stieglitz, et al., 2015). Reproductive cancers, such as endometrial, ovarian, breast and prostate cancers, are often associated with high levels of cumulative exposure to reproductive hormones, and appear to be rare among Tsimane as suggested by our clinical data. However other cancers of more infectious etiology, such as cervical cancer, are more common among Tsimane than in other populations (Stieglitz, et al., 2012).

Atherosclerosis, the main cause of cardiovascular disease (CVD), also appears to be largely absent among Tsimane for several reasons. First, obesity, high cholesterol, and hypertension are all associated with greater heart disease and stroke risk in Western countries. But the prevalences of adult obesity and hypercholesterolemia are much greater in the U.S. than among Tsimane (Gurven, et al., 2012). Even after adjusting for their lower body mass, rates of blood pressure increase in adulthood are lower among Tsimane than in 52 other populations from the INTERSALT study (Gurven, et al., 2012). Second, despite living in semi‐permanent villages with limited residential mobility, Tsimane are not sedentary. They engage in high levels of moderate physical activity (Gurven, Jaeggi, Kaplan, & Cummings, 2013) and show both strong cardiorespiratory fitness, as measured by VO_2_max (Pisor, Gurven, Blackwell, Kaplan, & Yetish, 2013), and a high prevalence of bradycardia (resting pulse <60). Third, Tsimane diet is lean and rich in fiber and omega‐3 fatty acids (Martin et al., 2012); also, it is uncommon for them to smoke cigarettes (Gurven, Kaplan, et al., 2009).

On the other hand, CRP and IL‐6, two inflammatory biomarkers that independently predict CVD morbidity and mortality, are elevated among Tsimane, likely as a result of pathogenic exposure (Blackwell et al., 2016; Gurven, et al., 2008). Tsimane high‐density lipoproteins (HDL) or “good cholesterol” levels are also low (Vasunilashorn et al., 2010). However, peripheral arterial disease (PAD), a precursor to fully developed artherosclerosis, as assessed by ankle brachial blood pressure index, is not observed among adults, although PAD increases with age in every other population studied to date (Gurven, Kaplan, et al., 2009). Several hundred “verbal autopsy” reports of recent and past deaths also reveal few cases of obvious cardiac or cerebrovascular events. Thus, mortality selection does not appear to be culling younger individuals with CVD. An ongoing collaboration with the *Horus* team (Thompson, Allam, Lombard, Wann, Sutherland, Sutherland, Al‐Tohamy Soliman, Frohlich, Mininberg, Monge, Vallodolid, Cox, Abd el‐Maksoud, Badr, Miyamoto, el‐Halim Nur el‐din, Narula, Finch, & Thomas, 2013) is quantifying levels of calcification in the coronary and thoracic aortic arteries based on high‐resolution computed tomography (CT) scans of older adults. Ongoing cranial CTs will assess changes in cerebral morphology to help understand cognitive aging, dementia, and the link between CVD and dementia. Given the relative absence of overt atherosclerosis and vascular disease, we expect to find lower rates of cognitive impairment and several types of cerebral atrophy in late adulthood among Tsimane than observed elsewhere. Alternatively, greater infection, inflammation, and limited schooling may accelerate cerebral atrophy, cognitive decline, and dementia.

One novel investigation currently underway is to explore the role of pathogenic exposure, particularly helminths, in risk of atherosclerosis and type 2 diabetes. Helminths, as part of their strategies to ensure their survival and reproduction, have multiple effects on their hosts. They consume blood lipids and glucose, alter lipid metabolism, and modulate immune function toward greater T_H_2 polarization. In combination, these conditions can lower blood cholesterol, reduce obesity, increase insulin sensitivity, decrease atheroma progression, and reduce the likelihood of atherosclerotic plaque rupture (Gurven, Trumble, Stieglitz, Blackwell, Michalik, Finch, & Kaplan, 2016; Wiria, Sartono, Supali, & Yazdanbakhsh, 2014). Consistent with these expectations, we have found that biomarkers of helminthic infection (e.g., IgE, eosinophils) are inversely associated with total cholesterol, LDL, HDL, and obesity (Vasunilashorn et al., 2010). Total cholesterol is almost 10 points lower among those with elevated CRP and IL‐6 and 19 points lower among those with elevated IgE controlling for potential confounders (Vasunilashorn et al., 2010).

In summary, Tsimane have a very low frequency of chronic diseases typically found in Western populations. The relative roles of energetic expenditure, diet, and pathogen burden in explaining differences in chronic disease risk among populations are still poorly understood due to the scarcity of detailed, longitudinal studies of appropriate populations in epidemiological transition, but each appears to play a contributory role. THLHP research shows that a more nuanced understanding of the role of infection‐induced inflammation in the etiology of chronic diseases is needed. Perhaps high levels of inflammation are atherogenic and diabetogenic only in the context of high adiposity and minimal exercise. Alternatively, different sources of inflammation may have different effects on chronic disease, with infection in some cases actually lowering chronic disease risk.

### 5.4 Production, cooperation, and sharing

The Tsimane production and sharing network is multigenerational. Total fertility rate is high (nine births per woman). Also, until late adolescence, Tsimane produce less food than they consume (Gurven, Stieglitz, Hooper, Gomes, & Kaplan, 2012; Hooper, Gurven, Winking, & Kaplan, 2015). Thus, the caloric burden on families can be substantial, especially for younger parents with multiple highly dependent offspring. Efficient food production peaks when Tsimane are in their 40s, especially for hunting and other difficult, skill‐intensive activities (Gurven, Winking, Kaplan, von Rueden, & McAllister, 2009) (Figure 5). Peak productivity extends long beyond peak strength (Gurven et al., 2006), suggesting the importance of skills‐based practice and learning. Although delayed productivity is clear for hunting (Gurven et al., 2006; Walker, Hill, Kaplan, & McMillan, 2002), expertise in a wide range of production, manufacturing, and other tasks (e.g., childcare, conflict mediation) is reported most frequently among middle‐aged or older adults (Schniter et al., 2015). Nuclear families provide much of the daily calories, with older adults, including parents, grandparents, and siblings providing substantial amounts of food to younger kin (Hooper, et al., 2015). As strength and functional ability decline in later adulthood, Tsimane shift emphasis toward low‐strength and high‐skill subsistence and political activities, including hook‐and‐line fishing and horticulture, conflict mediation, village leadership roles, and storytelling (Schniter et al., 2015). Food sharing is widespread within extended families, but more limited in scope than typically is described among foragers. Kinship and relative need, as determined by recipient age, productivity, family size, and health status largely determine the magnitude and direction of resource flows (Gurven, Stieglitz, et al., 2012; Hooper, et al., 2015).

Similar associations among food production, sharing behavior, and intergenerational transfers of calories to facilitate reproduction have been noted across human populations (Kaplan et al., 2000). These associations support two‐sex theoretical models of human longevity, emphasizing that postreproductive adults may “indirectly” reproduce by improving the survival and fertility of younger relatives (i.e., embodied capital model). Whereas among chimpanzees, our nearest primate relatives, reproductive decline is strongly linked to somatic decline and increasing risks of mortality, human reproductive senescence precedes somatic senescence by roughly 20‐25 years (Kaplan, Gurven, Winking, Hooper, & Stieglitz, 2010). After ceasing to reproduce, both men and women provide net economic transfers to children and grandchildren. On average, by the time they reach age 70, Tsimane rarely give food away to others outside of the household and so contribute less to sharing networks. The time delay between unproductivity due to physical deterioration and death appears to be short (Figure 5). Given this pattern of productivity and transfers, further delays in the age at menopause would produce net economic deficits within families because older adults would not be able to produce enough food for their own offspring (Kaplan et al., 2010).

Informal exchange networks help Tsimane manage multiple risks like sickness and injury, in addition to those from daily food shortfalls (Gurven, et al., 2012; Jaeggi, Hooper, Beheim, Kaplan, & Gurven, 2016). The “prices” implicitly negotiated in these informal exchange networks partly reflect individual differences in supply and demand, which itself relates to household needs and abilities (Jaeggi et al., 2016). Since the 1970s, increasing exposure to markets and state‐sponsored economic development initiatives have offered new ways for Tsimane to buffer against risks or shocks. Do greater market interactions substitute or supplement more traditional sharing networks? Somewhat surprisingly, those with greater wealth give a greater proportion of their food to others and have more sharing partners (Gurven et al., 2015). While villages with higher mean income do show less reciprocity, overall, our findings suggest that traditional sharing patterns are not eroded due to incipient market integration, a pattern that is at odds with reports from other populations (see Gurven et al., 2015, for discussion). Instead, sharing can be used for display and networking, in addition to buffering against risk. How Tsimane recuperate losses with aggregate shocks like the massive flood that affected over 40 villages in February 2014 is a subject of ongoing research.

In summary, the THLHP helps bridge theory with empirical data that establishes vital links between kin‐selected altruism, exchange networks, age profiles of productivity, and human life history traits. Food transfers are motivated by the interaction of genetic relatedness with need (Hooper, Gurven, et al., 2015). The needs of young children and families characterized by high dependency ratios predict downward transfers from older individuals, but only in proportion to shared genetic relatedness. These findings provide insights into how intergenerational transfers and increased longevity may have co‐evolved in a ratchet‐like process (Lee, 2008).

### 5.5 Marriage and reproductive decision‐making

The natural fertility regime of Tsimane provides opportunities to better understand costs of reproduction, as well as both determinants and consequences of reproductive decision‐making. A high fertility rate has existed at least since the 1950s in all areas of Tsimane territory, though menarche is earlier and interbirth intervals shorter closer to town (McAllister et al., 2012). Exclusive breastfeeding on demand occurs for at least four months, followed by mixed complementary feeding and breastfeeding for an additional year or until the next pregnancy (Martin, Garcia, Kaplan, & Gurven, 2016; Veile, Martin, McAllister, & Gurven, 2014). Such early complementary feeding of infants (earlier than World Health Organization recommendations of six months) seems to favor earlier resumption of ovulation at little or no cost to infant growth (Martin et al., 2016). These types of infant feeding decisions permit tests of parent‐offspring conflict given trade‐offs between child benefits and maternal residual reproduction. Indeed, ongoing research is addressing the extent to which birth order and birth spacing compromise children's health and nutritional status. An important tradeoff we have sought to understand is whether the direct energetic costs to mothers of high reproductive effort affect other life‐history allocations. Disposable soma theory proposes that investments in reproduction trade off against investments in maintenance and survival (Kirkwood, 1990). Maternal mortality due to pregnancy and childbirth complications is higher than that in most countries (Gurven et al., 2007). However, among survivors, evidence that cumulative costs of repeated pregnancies diminish women's health and nutritional status is mixed (Gurven, Costa, Trumble, Stieglitz, Beheim, Eid Rodriguez, Hooper, & Kaplan, 2016). Although weight loss and anemia are common shortly after birth, there is minimal evidence of compromised health and nutritional status over cumulative births. Repeated births seem only to be associated with a greater likelihood of cystocele (bladder prolapse) and osteopenia (Stieglitz, Beheim, et al., 2015). Ongoing research is addressing potential short‐ and long‐term costs to reproduction among both parents, given substantial contributions to the energetics of reproduction and linked fertility schedules of men and women.

The “ideal” family size reported by Tsimane women and men is lower than their actual fertility. Spouses’ ideal fertility preferences converge among more educated couples living near town, who generally report ideal family sizes of three or four children (McAllister et al., 2012). However, individuals reporting the smallest ideal family sizes actually have the highest observed fertility. One potential explanation is that schooling in the absence of viable wage opportunities hinders the incentives to reduce fertility that typically accompany greater human capital investment. Instead, status still derives largely from one's traditional social networks and, by extension, larger families. Consistent with this view, female role models are reported to be those with the largest families (McAllister et al., 2012). The current cohort of young adult women, however, is delaying their first births more than previous generations did (Kaplan, Hooper, Stieglitz, & Gurven, 2015). It appears that a nascent fertility transition in some areas of the Tsimane territory is underway. Understanding the social, economic, and psychological determinants of fertility change is critical given Tsimane concerns over limited land, low meat supplies, and growing demand for market goods.

Tsimane marriages are largely monogamous; <5% of marriages are polygynous. Polygynous marriages mostly involve sisters, but their dynamics and implications are relatively unexplored. While polygyny generates higher fertility for husbands, it is associated with lower fertility for second wives (Winking, Stieglitz, Kurten, Kaplan, & Gurven, 2013). In general, marriages are relatively stable, with divorce uncommon after the birth of children. Extramarital sexual relationships by husbands are most likely to occur early in marriage rather than later, when wives’ reproductive value is low and offspring dependency is high (Winking, Kaplan, Gurven, & Rucas, 2007). This is consistent with the view of pair bonds functioning to provision children. Men's and women's fertility schedules are strongly linked in marriage: 90% of men whose wives reached menopause did not reproduce again after their wife's last birth, consistent with an “effective menopause” or male reproductive cessation (Kaplan et al., 2010). Of the 10% who did reproduce, half (5.2%) were polygynously married and had a child with a younger co‐wife.

Market integration brings new social risks and costs. Men's wage labor offers opportunities and benefits but, as observed elsewhere, is also associated with men's absenteeism, extramarital affairs, domestic violence, and sexually transmitted infection (Stieglitz, et al., 2012; Stieglitz, Kaplan, Gurven, Winking, & Tayo, 2011). Current evidence shows that spousal conflict over the use of fungible and liquid cash results from men's attempts to increase personal benefits at a cost to the family (e.g., through pursuit of extramarital affairs, often in town or other villages) (Stieglitz, Gurven, Kaplan, & Winking, 2012; Stieglitz et al., 2011). Over 85% of Tsimane women report experiencing some form of physical abuse in marriage. Men may use physical violence i.e., to quell women's objections to male disinvestment, maintain women's parental investment, and dissuade women from pursuing sexual relationships with other men. The likelihood of reporting physical wife abuse is greatest in marriages when the husband has recently had an extramarital affair (Stieglitz, et al., 2012). Greater market integration and access to perfectly fungible and liquid resources can therefore disrupt patterns of reciprocity within and across households.

In summary, the importance of resource transfers and cooperation in Tsimane life history is revealed by the conflicts of interests that arise within and among families. Male economic transfers to women and children are important, but can also be a source of tension between husbands and wives when expectations of paternal investment are unmet. The majority of verbal conflict between spouses and men's violence against women stems from complaints about lack of paternal investment, especially the diversion of resources to women other than the man's wife. These findings suggest a revision of current debates about the significance of male parental investment within marriage. In the Tsimane context, men provide the majority of calories that families consume, but men's interests are not aligned only with those of their families. Men often invest in extramarital sexual relationships, especially as young adults; this is a source of great conflict within marriages. As children are produced in the marriage and both partners age, these conflicts subside, perhaps because as the family grows there are more mouths to feed, increasing the value of male investment and the costs of disinvestment.

### 5.6 Social status, personality, and determinants of reproductive success

The THLHP has also advanced the study of social status, an immaterial positional good of psychological importance in all social primates, by focusing on its determinants and benefits. Among men, dominance, or the ability to win dyadic physical confrontations, is associated with higher intramarital fertility for age, while prestige, the gaining of respect and influence from expertise or character, provides both higher intramarital fertility and lower offspring mortality; both are associated with a greater number of extramarital affairs (von Rueden, Gurven, & Kaplan, 2011). Both types of status elicit support from allies and deference from competitors, but high‐status men are not provisioned more than their peers. Prestigious but not dominant men marry wives who first give birth at earlier ages, which multivariate analysis suggests is the strongest pathway between status and fitness among Tsimane. Men's prestige derives from leadership abilities, oratory skill, traditional productive skills like hunting, and novel skills that are crucial for obtaining market resources and navigating town life. High‐status men have lower levels of the stress hormone cortisol and are less likely to suffer from respiratory illness (von Rueden, Trumble, Thompson, Stieglitz, Hooper, Blackwell, Kaplan, & Gurven, 2014).

Women's status competition is less overt but equally important. The domains of conflict shift across the life course (Rucas, Gurven, Winking, & Kaplan, 2012). Although physical attractiveness is a key factor in studies of women's status in Western populations, other characteristics, such as work ethic, trustworthiness, communication abilities, and childcare abilities, can trump its importance in evaluations of Tsimane women's status (Rucas, Gurven, Kaplan, Winking, Gangestad, & Crespo, 2006b). Subjects of ongoing research include the costs and benefits of women's status investments, changes in their status with greater schooling and market integration, and how men's and women's status interrelate and affect pair‐bond dynamics (e.g., bargaining) and child wellbeing.

Personality is another immaterial individual trait of profound importance in urban populations, but is rarely studied in small‐scale human societies. Our exploration of the origins and implications of personality differences among Tsimane has provided novel insights. First, many personality dimensions are associated with fertility. For men, effects appear to be uniform across all areas of Tsimane territory, whereas effects for women vary by geographic proximity to town (Gurven, et al., 2014). These findings support balancing or fluctuating selection models geared to explain why heritable variation in personalities seems to be maintained over time, despite selection gradients suggesting that certain personality types have higher fitness than others. Some aspects of Tsimane personality appear to be adaptively calibrated to phenotypic condition. For both men and women, extraversion and prosocial orientation are associated with several types of embodied capital, including body size, physical strength, schooling, and fluency in Spanish (von Rueden, Lukaszewski, & Gurven, 2015). Thus, the high (“hidden”) heritability of personality may be due in part to heritability of various components of embodied capital.

In summary, status and personality differences have fitness consequences even in “egalitarian” societies. Via direct and indirect routes, dominance and prestige are different forms of male status that are linked to reproductive outcomes. One route may be through increased access to younger fecund women; another may be through increased access to hard‐working women with higher fertility; and a third may be through increased resource access and generosity, also resulting in higher marital fertility. Allies and social support are both causes and effects of high status and may be critical as a form of insurance against food shortfalls, conflicts, and other risks. Understanding how social status is achieved and inherited, the types of investments that help build social capital, and trade‐offs with other forms of embodied capital in both sexes is an area of ongoing research.

### 5.7 Modernization and socioeconomic change

The THLHP provides insight into the complex relationships between modernization and behavior, lifestyle, and health. The transition in Tsimane lifestyle and environment is highly variable throughout Tsimane territory. Natural variation in modernizing influences creates a unique quasi‐experimental condition for exploring effects of market integration and concomitant changes in diet, physical activity (Gurven, et al., 2013), reproductive strategies, and access to health care on health (Stieglitz, et al., 2012), fertility (Kaplan et al., 2015; McAllister et al., 2012), risk management strategies, social behavior (Gurven, 2004b; Gurven et al., 2015), and cognitive performance (Gurven, et al., 2016). Changes over the past decade of THLHP surveillance will provide additional opportunities for understanding how socioeconomic change occurs and why only some individuals pursue market‐related opportunities. For example, extraverted adults are more likely to live near town, be fluent in Spanish, and obtain schooling, suggesting some self‐selection in migration and market exposures (von Rueden et al., 2015). The low level of market involvement by Tsimane does not appear to disrupt systems of exchange and downward intergenerational transfers (Gurven et al., 2015). However, it does provide men with money and often requires their absence from family to engage in wage labor or buy and sell market goods. This can increase spousal conflict and violence through its effects on extramarital affairs, reduced subsistence efforts, and alcohol consumption (Stieglitz, et al., 2012; Stieglitz et al., 2011). Market involvement is also associated with increased access to sugar, oil, and other concentrated forms of calories. These changes are likely to affect chronic disease risk, as anecdotally shown by higher blood sugar levels among the Tsimane political leadership, who tend to live sedentary, urbanized lifestyles.

## 6 Conclusion

The THLHP has produced exciting findings of broad relevance to evolutionary anthropology and contributed to critical debates in the social and life sciences. Though pitched originally to improve our understanding of human life history evolution, its scope has expanded to include a broad range of topics regarding health, socioecology, and culture. This was possible only with joint directorship, engaged interdisciplinary and international colleagues, and our excellent team of postdoctoral scholars, graduate students, and THLHP personnel. Continuous funding of large‐scale research is a substantial challenge, but we believe that the payoffs in terms of scholarship and direct community benefits make this a model worth emulating elsewhere. It has been our hope that the THLHP helps make evolutionary anthropology more salient within the broader social and biological sciences. An ethnographic emphasis combined with an evolutionary and ecological focus can influence multiple disciplines by highlighting aspects of human diversity typically ignored by more mainstream research traditions. For example, Tsimane findings support new ways of thinking about the role of inflammation on chronic disease, the potential benefits of helminth infection, implications of a sexual division of labor, and functional significance of relatively stable personality traits. THLHP collaborations with nonanthropologists help bring the strengths of a holistic evolutionary anthropology into other fields.

Although the Tsimane represent a single case study, an important goal is to stimulate more cross‐cultural comparative research using standardized methods. For example, we recognize that a comparative approach will be needed to make broader inferences about the relative importance of infection, lifestyle factors, and genetics in chronic disease morbidity (Box 1). In this case, comparison of populations varying in parasite burden, diet, and activity profile can provide insight into the role of immune dysregulation on chronic disease progression. Coordination with established field sites and researchers can be a critical first step in this direction. We can provide methodological protocols to help standardize methods across field sites.

## 7 Data and protocol availability

De‐identified data have been published as supplements in many of our publications. Appropriate requests for specific data have also been honored. Plans are currently underway to clean and organize datasets for restricted public access given federal guidelines set by NIH and NSF. These will be made available via a data portal on our website: www.unm.edu/~tsimane.

## Glossary

**Atherosclerosis**—A disease in which plaque, composed of fat, cholesterol, calcium and other substances, builds up within the arteries. Over time, plaque hardens, narrowing the arteries and limiting blood flow to organs and other body parts. Atherosclerosis can affect any artery and lead to heart attack, stroke, or death.

**Benign prostate hyperplasia**—A condition in men in which the prostate gland is enlarged but not cancerous. Enlargement constricts the urethra and compresses the bladder, weakening the bladder's ability to empty completely. Urethra narrowing and urinary retention in the bladder lead to a variety of health problems, including trouble urinating or frequent, painful urination.

**Cumulative culture**—The accumulation over time of trait modifications obtained primarily through social learning. Modifications are often ratchet‐like, leading to the development and maintenance of complex technologies.

**Disposable soma theory**—The optimal energy allocation to growth, metabolism, reproduction, and repair that maximizes fitness often results in compromised allocation to maintenance, resulting in a physical body that deteriorates with age. Given the declining force of selection with age, the theory proposes that natural selection optimizes levels of somatic repair below that required for extensive longevity due to prioritized fitness gains of earlier investments in reproduction.

**Embodied capital theory**—A model that considers extension of human life span as a coordinated response to a dietary shift focused on a skill‐intensive diet with food sharing. Delayed juvenility and dependency, combined with a learning‐intensive cultural niche early in life, are subsidized through large economic surpluses in adulthood, multigenerational sharing, and biparental care.

**Evolutionary mismatch**—A state of disequilibrium whereby advantageous traits that evolved in one environment become maladaptive in new environments. Rapid, drastic changes in human environments and lifestyles over recent millennia, especially the past 200 years, outpace the ability of genetic changes to adapt organisms to the newer environment, leading to mismatched disease and behavior.

**Exogenous (extrinsic) mortality**—Mortality from external causes (e.g., accidental injuries, pathogen exposure), which are distinguished from internal or intrinsic causes (e.g., degenerative disease, genetic susceptibility). Extrinsic mortality is often described as being beyond the control of an organism to alter, but organisms often exert some control to reduce mortality risks, e.g., by altering patterns of movement to avoid accidents or investing more energy in immune function.

**Fluid versus crystallized cognitive abilities**—Fluid intelligence is the ability to problem‐solve and reason in novel situations, whereas crystallized intelligence is the ability to remember acquired knowledge and learned skills. Some related abilities such as processing speed and working memory may not neatly fit the classical “fluid” versus “crystallized” distinction.

**Grandmother hypothesis**—Model of extended postreproductive human life span emphasizing the inclusive fitness contributions of grandmothers, primarily through increasing survival of grandoffspring and daughter fertility.

**Helminths**—Multicellular eukaryotic invertebrates with tube‐like or flattened bodies exhibiting bilateral symmetry. Many helminths are free‐living organisms in aquatic and terrestrial environments. Most inhabit the intestines (e.g., roundworm, hookworm), but some inhabit blood vessels (e.g. schistosomes)

**Horticulturalists**—Use hand tools to grow domesticated plants. Horticulturalists do not use draft animals, irrigation, specially prepared fertilizers, or machinery. Horticulturalists often supplement their subsistence efforts through other means including hunting, fishing, or foraging.

**Inflammation**—The body's initial protective response to harmful stimuli (e.g., pathogens, irritants, damaged cells), which involves immune cells, blood vessels, and molecular mediators. Inflammation helps eliminate the initial cause of cell and tissue injury, clear out damaged cells and tissues, and initiate cell and tissue repair. Though inflammation is necessary for survival, chronic inflammation contributes to aging‐related diseases such as heart disease and diabetes.

**Intergenerational transfers**—Flows of material (e.g., food) or nonmaterial (e.g., information) resources across generations, e.g., from parents to offspring or grandparents to grandoffspring. Intergenerational transfers have a central role in several theories of human life history evolution. While resources are also transferred “upward” in humans, from younger to older generations, the net flow of resources in small‐scale subsistence economies is from older to younger generations.

**Skills‐intensive foraging niche**—Human forager diets consist largely of foods requiring high levels of skill and strength. Meat and other important foods (e.g., tubers, larvae, honey, nuts) require extraction from a substrate (often with technology), intensive processing, and assistance from others. Hunting return rates more than double from ages 20 to 40 years, even though strength peaks in the mid‐20s. Delayed peak efficiency relative to peak strength has also been documented for other foods. Thus, large caloric deficits are incurred early in life, requiring transfers from others, and only by the mid to late teens do individuals start producing more calories than they consume. Surplus caloric production increases with age, peaks in the 40s, then slowly declines until reaching consumption levels by the 70s.

## References

[evan21515-bib-0001] Barrett, H. C. , Bolyanatz, A. , Crittenden, A. N. , Fessler, D. M. , Fitzpatrick, S. , Gurven, M. , Henrich, J. , Kanovsky, M. , Kushnick, G. , Pisor, A. , Scelza, B.A. , Stich, S. , von Rueden, C. , Wanying, Z. , & Laurence S. (2016). Small‐scale societies exhibit fundamental variation in the role of intentions in moral judgment. Proceedings of the National Academy of Sciences, 113, 4688–4693. 10.1073/pnas.1522070113 PMC4855604 27035959

[evan21515-bib-0002] Blackwell, A. D. , Gurven, M. D. , Sugiyama, L. S. , Madimenos, F. C. , Liebert, M. A. , Martin, M. A. , Kaplan, H.S. , & Snodgrass, J. J. (2011). Evidence for a peak shift in a humoral response to helminths: Age Profiles of IgE in the Shuar of Ecuador, the Tsimane of Bolivia, and the U.S. NHANES. PLoS Negl Trop Dis, 5, e1218. doi:10.1371/journal.pntd.0001218 21738813 10.1371/journal.pntd.0001218 PMC3125146

[evan21515-bib-0003] Blackwell, A. D. , Martin, M. , Kaplan, H. , & Gurven, M. (2013). Antagonism between two intestinal parasites in humans: the importance of co‐infection for infection risk and recovery dynamics. Proceedings of the Royal Society of London B: Biological Sciences, 280(1769), 20131671. 10.1098/rspb.2013.1671 PMC3768312 23986108

[evan21515-bib-0004] Blackwell, A. D. , Tamayo, M. A. , Beheim, B. , Trumble, B. C. , Stieglitz, J. , Hooper, P. L. , Martin, M. , Kaplan, H. , & Gurven, M. (2015). Helminth infection, fecundity, and age of first pregnancy in women. Science, 350(6263), 970–972. 26586763 10.1126/science.aac7902 PMC5953513

[evan21515-bib-0005] Blackwell, A. D. , Trumble, B. C. , Maldonado Suarez, I. , Stieglitz, J. , Beheim, B. A. , Snodgrass, J. J. , Kaplan, H. , & Gurven, M. (2016). Immune function in Amazonian horticulturalists. Annals of Human Biology, 43, 382–396. doi:10.1080/03014460.2016.1189963 27174705 10.1080/03014460.2016.1189963 PMC4976077

[evan21515-bib-0006] Blackwell, A. D. , Urlacher, S. S. , Beheim, B. A. , von Rueden, C. , Jaeggi, A. V. , Stieglitz, J. , Trumble, B.C. , Gurven, M. , & Kaplan, H. (2016). Growth references for Tsimane forager‐horticulturalists of the Bolivian Amazon. American Journal of Physical Anthropology, doi:10.1002/ajpa.23128. 10.1002/ajpa.23128 PMC5321633 28218400

[evan21515-bib-0007] Blurton Jones, N. (2016). Demography and Evolutionary Ecology of Hadza Hunter‐Gatherers (Vol.71): Cambridge University Press.

[evan21515-bib-0008] Borgerhoff Mulder, M. , Bowles, S. , Hertz, T. , Bell, A. , Beise, J. , Clark, G. , Fazzio, I. , Gurven, M. , Hill, K. , Hooper, P.L. , Irons, W. , Kaplan, H. , Leonetti, D. , Low, B. , Marlowe, F. , McElreath, R. , Naidu, S. , Nolin, D. , Piraino, P. , Quinlan, R. , Schniter, E. , Sear, R. , Shenk, M. , Smith, E.A. , von Rueden, C. , & Wiessner, P. (2009). Intergenerational Wealth Transmission and the Dynamics of Inequality in Small‐Scale Societies. Science, 326(5953), 682. 19900925 10.1126/science.1178336 PMC2792081

[evan21515-bib-0009] Fessler, D. M. , Barrett, H. C. , Kanovsky, M. , Stich, S. , Holbrook, C. , Henrich, J. , Bolyanatz, A.H. , Gervais, M.M. , Gurven, M. , Kushnick, G. , Pisor, A.C. , von Rueden, C. , & Laurence, S. F (2015). Moral parochialism and contextual contingency across seven societies. Paper presented at the Proc. R. Soc. B. 10.1098/rspb.2015.0907 PMC4632614 26246545

[evan21515-bib-0010] Fumagalli, M. , Sironi, M. , Pozzoli, U. , Ferrer‐Admettla, A. , Pattini, L. , & Nielsen, R. (2011). Signatures of environmental genetic adaptation pinpoint pathogens as the main selective pressure through human evolution. PLoS Genet, 7, e1002355. 22072984 10.1371/journal.pgen.1002355 PMC3207877

[evan21515-bib-0011] Glowacki, L. , & von Rueden, C. (2015). Leadership solves collective action problems in small‐scale societies. Phil. Trans. R. Soc. B, 370(1683), 20150010. 26503683 10.1098/rstb.2015.0010 PMC4633846

[evan21515-bib-0012] Gurven, M. (2004a). Does market exposure affect economic behavior? The ultimatum game and public goods game among the Tsimane' of Bolivia In Henrich J., Boyd R., Bowles S., Gintis H., Fehr E., & Camerer C. (Eds.), Foundations of Human Sociality: Ethnography and Experiments in 15 Small‐Scale Societies (pp. 194 ‐ 231). Oxford: Oxford University Press.

[evan21515-bib-0013] Gurven, M. (2004b). Economic games among the Amazonian Tsimane: Exploring the roles of market access, costs of giving, and cooperation on pro‐social game behavior. Experimental Economics, 7, 5–24.

[evan21515-bib-0014] Gurven, M. (2012a). Human survival and life history in evolutionary perspective The evolution of primate societies (pp. 293–314).

[evan21515-bib-0015] Gurven, M. (2012b). Infant and fetal mortality among a high fertility and mortality population in the Bolivian Amazon. Social Science & Medicine, 75, 2493–2502. doi:https://doi.org/10.1016/j.socscimed.2012.09.030 23092724 10.1016/j.socscimed.2012.09.030 PMC3502712

[evan21515-bib-0016] Gurven, M. , Blackwell, A. D. , Rodríguez, D. E. , Stieglitz, J. , & Kaplan, H. (2012). Does blood pressure inevitably rise with age? Longitudinal evidence among forager‐horticulturalists. Hypertension, 60, 25–33. 22700319 10.1161/HYPERTENSIONAHA.111.189100 PMC3392307

[evan21515-bib-0017] Gurven, M. , Costa, M. , Trumble, B. , Stieglitz, J. , Beheim, B. , Eid Rodriguez, D. , Hooper, P.L. , & Kaplan, H. (2016). Health costs of reproduction are minimal despite high fertility, mortality and subsistence lifestyle. Nature Scientific Reports, 6. doi:10.1038/srep30056 10.1038/srep30056 PMC4951795 27436412

[evan21515-bib-0018] Gurven, M. , & Fenelon, A. (2009). Has actuarial aging “slowed” over the past 250 years? A comparison of small‐scale subsistence populations and european cohorts. Evolution, 63(4), 1017–1035. 19220451 10.1111/j.1558-5646.2008.00592.x PMC3390018

[evan21515-bib-0019] Gurven, M. , Fuerstenberg, E. , Trumble, B. C. , Stieglitz, J. , Beheim, B. , Davis, H. , & Kaplan, H. (2016). Cognitive performance across the life course of Bolivian forager‐farmers with limited schooling. Developmental Psychology, doi:10.1037/dev0000175 10.1037/dev0000175 PMC5191915 27584668

[evan21515-bib-0020] Gurven, M. , Jaeggi, A. V. , Kaplan, H. , & Cummings, D. (2013). Physical activity and modernization among Bolivian Amerindians. PLoS ONE, 8, e55679. 23383262 10.1371/journal.pone.0055679 PMC3561330

[evan21515-bib-0021] Gurven, M. , Jaeggi, A. V. , von Rueden, C. , Hooper, P. L. , & Kaplan, H. (2015). Does market integration buffer risk, erode traditional sharing practices and Increase Inequality? A test among Bolivian forager‐farmers. Human Ecology, 43, 515–530. 10.1007/s10745-015-9764-y PMC4624453 26526638

[evan21515-bib-0022] Gurven, M. , & Kaplan, H. (2007). Longevity among hunter‐gatherers: a cross‐cultural comparison. Population and Development Review, 33, 321–365.

[evan21515-bib-0023] Gurven, M. , & Kaplan, H. (2008). Beyond the grandmother hypothesis: evolutionary models of human longevity. The cultural context of aging: worldwide perspectives (Vol.3), pp. 53–66).

[evan21515-bib-0024] Gurven, M. , Kaplan, H. , Crimmins, E. , Finch, C. , & Winking, J. (2008). Lifetime inflammation in two epidemiological worlds: the Tsimane of Bolivia and the United States. Journal of Gerontology Biological Sciences, 63 *A*, 196–199. 10.1093/gerona/63.2.196 PMC2952348 18314457

[evan21515-bib-0025] Gurven, M. , Kaplan, H. , & Gutierrez, M. (2006). How long does it take to become a proficient hunter? Implications for the evolution of delayed growth. Journal of Human Evolution, 51, 454–470. 16797055 10.1016/j.jhevol.2006.05.003

[evan21515-bib-0026] Gurven, M. , Kaplan, H. , Winking, J. , Eid, D. , Vasunilashorn, S. , Kim, J. , Finch, C. , & Crimmins, E. (2009). Inflammation and infection do not promote arterial aging and cardiovascular disease among lean Tsimane forager‐horticulturalists. PLoS ONE, 4, e6590. doi:10.1371/journal.pone.0006590 19668697 10.1371/journal.pone.0006590 PMC2722089

[evan21515-bib-0027] Gurven, M. , Kaplan, H. , & Zelada Supa, A. (2007). Mortality experience of Tsimane Amerindians: regional variation and temporal trends. American Journal of Human Biology, 19, 376–398. 17421012 10.1002/ajhb.20600

[evan21515-bib-0028] Gurven, M. , Mulder, M. B. , Hooper, P. L. , Kaplan, H. , Quinlan, R. , Sear, R. , Hertz, T. (2010). Domestication alone does not lead to inequality. Current Anthropology, 51(1), 49–64.

[evan21515-bib-0029] Gurven, M. , Stieglitz, J. , Hooper, P. L. , Gomes, C. , & Kaplan, H. (2012). From the womb to the tomb: The role of transfers in shaping the evolved human life history. Experimental Gerontology, 47, 807–813. 22595699 10.1016/j.exger.2012.05.006 PMC3437008

[evan21515-bib-0030] Gurven, M. , Trumble, B. , Stieglitz, J. , Blackwell, A. , Michalik, D. , Finch, C. , & Kaplan, H. (2016). Cardiovascular disease and type 2 diabetes in evolutionary perspective: a critical role for helminths? Evolution, medicine, and public health, 1, 338–357. 10.1093/emph/eow028 PMC5101910 27666719

[evan21515-bib-0031] Gurven, M. , & Von Rueden, C. (2006). Hunting, social status and biological fitness. *Biodemography and* Social Biology, 53(1‐2), 81–99. 21516952 10.1080/19485565.2006.9989118

[evan21515-bib-0032] Gurven, M. , von Rueden, C. , Massenkoff, M. , Kaplan, H. , & Lero Vie, M. (2013). How universal is the Big Five? Testing the five‐factor model of personality variation among forager–farmers in the Bolivian Amazon. Journal of Personality and Social Psychology, 104(2), 354. 23245291 10.1037/a0030841 PMC4104167

[evan21515-bib-0033] Gurven, M. , von Rueden, C. , Stieglitz, J. , Kaplan, H. , & Rodriguez, D. E. (2014). The evolutionary fitness of personality traits in a small‐scale subsistence society. Evolution and Human Behavior, 35, 17–25. 10.1016/j.evolhumbehav.2013.09.002 PMC3885165 24415896

[evan21515-bib-0034] Gurven, M. , & Winking, J. (2008). Collective action in action: Prosocial behavior in and out of the laboratory. American Anthropologist, 110(2), 179–190.

[evan21515-bib-0035] Gurven, M. , Winking, J. , Kaplan, H. , von Rueden, C. , & McAllister, L. (2009). A bioeconomic approach to marriage and the sexual division of labor. Human Nature, 20, 151–183. 25526956 10.1007/s12110-009-9062-8 PMC5486514

[evan21515-bib-0036] Gurven, M. , Zanolini, A. , & Schniter, E. (2008). Culture sometimes matters: Intra‐cultural variation in pro‐social behavior among Tsimane Amerindians. Journal of Economic Behavior & Organization, 67, 587–607. 19122839 10.1016/j.jebo.2007.09.005 PMC2582818

[evan21515-bib-0037] Gurven, M. D. (2014). The Tsimane'Rarely Punish: An Experimental Investigation of Dictators, Ultimatums, and Punishment Experimenting with Social Norms: Fairness *and* Punishment in Cross‐Cultural Perspective (pp. 197).

[evan21515-bib-0038] Gurven, M. D. , Trumble, B. C. , Stieglitz, J. , Blackwell, A. D. , Michalik, D. E. , Finch, C. E. , & Kaplan, H. S. (2016b). Cardiovascular disease and type 2 diabetes in evolutionary perspective: A critical role for helminths? Evolution, medicine, and public health, 2016(1), 338–357. doi:10.1093/emph/eow028 10.1093/emph/eow028 PMC5101910 27666719

[evan21515-bib-0039] Gurven, M. D. , Trumble, B. C. , Stieglitz, J. , Yetish, G. , Cummings, D. , Blackwell, A. D. , Beheim, B. , Kaplan, H.S. , & Pontzer, H. (2016). High resting metabolic rate among Amazonian forager‐horticulturalists experiencing high pathogen burden. American Journal of Physical Anthropology, 161(3), 414–425. 27375044 10.1002/ajpa.23040 PMC5075257

[evan21515-bib-0040] Han, C. , Martin, M. , Dichosa, A. , Daughton, A. , Frietze, S. , Kaplan, H. , Gurven, M.D. , & Alcock, J. (2016). Salivary microbiomes of indigenous Tsimane mothers and infants are distinct despite frequent premastication. PeerJ, 4, e2660. 27833819 10.7717/peerj.2660 PMC5101600

[evan21515-bib-0041] Hawkes, K. (2003). Grandmothers and the evolution of human longevity. American Journal of Human Biology, 15, 380–400. 12704714 10.1002/ajhb.10156

[evan21515-bib-0042] Hawks, J. , Wang, E. T. , Cochran, G. M. , Harpending, H. C. , & Moyzis, R. K. (2007). Recent acceleration of human adaptive evolution. Proceedings of the National Academy of Sciences, USA, 104, 20753–20758. doi:10.1073/pnas.0707650104 10.1073/pnas.0707650104 PMC2410101 18087044

[evan21515-bib-0043] Henn, B. M. , Cavalli‐Sforza, L. L. , & Feldman, M. W. (2012). The great human expansion. Proceedings of the National Academy of Sciences, 109, 17758–17764. doi:10.1073/pnas.1212380109 10.1073/pnas.1212380109 PMC3497766 23077256

[evan21515-bib-0044] Henrich, J. , Boyd, R. , Bowles, S. , Camerer, C. , Fehr, E. , Gintis, H. , McElreath, R. , Alvard, M. , Barr, A. , Ensminger, J. , Henrich, N.S. , Hill, K. , Gil‐White, F. , Gurven, M. , Marlowe, F.W. , Patton, J.Q. , & Tracer, D. (2005). “Economic man” in cross‐cultural perspective: Behavioral experiments in 15 small‐scale societies. Behavioral and Brain Sciences, 28(06), 795–815. doi:10.1017/S0140525X05000142 16372952 10.1017/S0140525X05000142

[evan21515-bib-0045] Henrich, J. , Ensminger, J. , McElreath, R. , Barr, A. , Barrett, C. , Bolyanatz, A. , Cardenas, J.C. , Gurven, M. , Gwako, E. , Henrich, N. , Lesorogol, C. , Marlowe, F. , Tracer, D. , & Ziker, J. (2010). Markets, religion, community size, and the evolution of fairness and punishment. Science, 327(5972), 1480–1484. 20299588 10.1126/science.1182238

[evan21515-bib-0046] Henrich, J. , McElreath, R. , Barr, A. , Ensminger, J. , Barrett, C. , Bolyanatz, A. , Cardenas, J.C. , Gurven, M. , Gwako, E. , Henrich, N. , Lesorogol, C. , Marlowe, F. , Tracer, D. , & Ziker, J. (2006). Costly Punishment Across Human Societies. Science, 312(5781), 1767–1770. 16794075 10.1126/science.1127333

[evan21515-bib-0047] Hill, K. , Barton, M. , & Hurtado, A. M. (2009). The emergence of human uniqueness: Characters underlying behavioral modernity. Evolutionary Anthropology 18, 187–200.

[evan21515-bib-0048] Hill, K. , & Hurtado, A. M. (1996). Ache Life History: the ecology and demography of a foraging people. New York: Aldine de Gruyter.

[evan21515-bib-0049] Hodges‐Simeon, C. R. , Gurven, M. , Cárdenas, R. A. , & Gaulin, S. J. (2013). Voice change as a new measure of male pubertal timing: a study among Bolivian adolescents. Annals of Human Biology, 40(3), 209–219. 23388046 10.3109/03014460.2012.759622

[evan21515-bib-0050] Hodges‐Simeon, C. R. , Gurven, M. , & Gaulin, S. J. (2015). The low male voice is a costly signal of phenotypic quality among Bolivian adolescents. Evolution and Human Behavior, 36(4), 294–302.

[evan21515-bib-0051] Hodges‐Simeon, C. R. , Gurven, M. , Puts, D. A. , & Gaulin, S. J. (2014). Vocal fundamental and formant frequencies are honest signals of threat potential in peripubertal males. Behavioral Ecology, aru081. 10.1093/beheco/aru081 PMC4095947 25024638

[evan21515-bib-0052] Hodges‐Simeon, C. R. , Sobraske, K. N. H. , Samore, T. , Gurven, M. , & Gaulin, S. J. (2016). Facial Width‐To‐Height Ratio (fWHR) Is Not Associated with Adolescent Testosterone Levels. PLoS ONE, 11(4), e0153083. 27078636 10.1371/journal.pone.0153083 PMC4831733

[evan21515-bib-0053] Hooper, P. , Gurven, M. , & Kaplan, H. (2014). Social and Economic Underpinnings of Human Biodemography Advances in Biodemography: Cross‐Species Comparisons of Social Environments and Social Behaviors, and their Effects on Health and Longevity. Washington, D.C: National Academy Press.

[evan21515-bib-0054] Hooper, P. L. , DeDeo, S. , Caldwell Hooper, A. E. , Gurven, M. , & Kaplan, H. S. (2013). Dynamical structure of a traditional Amazonian social network. Entropy, 15(11), 4932–4955. 25053880 10.3390/e15114932 PMC4104206

[evan21515-bib-0055] Hooper, P. L. , Demps, K. , Gurven, M. , Gerkey, D. , & Kaplan, H. S. (2015). Skills, division of labour and economies of scale among Amazonian hunters and South Indian honey collectors. Philosophical Transactions of the Royal. Society of London. Series B, 370, 20150008. 26503681 10.1098/rstb.2015.0008 PMC4633844

[evan21515-bib-0056] Hooper, P. L. , Gurven, M. , Winking, J. , & Kaplan, H. S. (2015). Inclusive fitness and differential productivity across the life course determine intergenerational transfers in a small‐scale human society. Proceedings of the Royal Society of London B: Biological Sciences, 282, 20142808. 10.1098/rspb.2014.2808 PMC4345452 25673684

[evan21515-bib-0057] Horvath, S. , Gurven, M. , Levine, M. E. , Trumble, B. C. , Kaplan, H. , Allayee, H. , Ritz, B.R. , Chen, B. , Lu, A.T. , Rickbaugh, T.M. , Jamieson, B.D. , Sun, D. , Li, S. , Chen, W. , Quintana‐Murci, L. , Fagny, M. , Kobor, M. , Tsao, P.S. , Reiner, A.P. , Edlefsen, K.L. , Absher, D. , & Assimes, T. (2016). An epigenetic age analysis of race/ethnicity, gender and coronary heart disease addresses several paradoxes surrounding mortality. Genome Biology, 17, 171. 27511193 10.1186/s13059-016-1030-0 PMC4980791

[evan21515-bib-0058] Howell, N. (1979). Demography of the Dobe! Kung. New York: Academic Press.

[evan21515-bib-0059] Howell, N. (2010). Life histories of the Dobe! Kung: food, fatness, and well‐being over the life span (Vol. 4): Univ of California Press.

[evan21515-bib-0060] INE. (2012). Bolivia Características de Población y Vivienda: Censo Nacional de Población y Vivienda 2012. Retrieved from La Paz:

[evan21515-bib-0061] Jaeggi, A. V. , & Gurven, M. (2013). Reciprocity explains food sharing in humans and other primates independent of kin selection and tolerated scrounging: a phylogenetic meta‐analysis. Proceedings of the Royal Society B: Biological Sciences, 280(1768). 10.1098/rspb.2013.1615 PMC3757985 23945693

[evan21515-bib-0062] Jaeggi, A. V. , Hooper, P. L. , Beheim, B. , Kaplan, H. , & Gurven, M. (2016). Reciprocal exchange patterned by market forces helps explain cooperation in a small‐scale society. Current Biology, 26, 2180–2187. 27451903 10.1016/j.cub.2016.06.019

[evan21515-bib-0063] Jaeggi, A. V. , Trumble, B. C. , Kaplan, H. S. , & Gurven, M. (2015). Salivary oxytocin increases concurrently with testosterone and time away from home among returning Tsimane'hunters. Biology letters, 11(3), 20150058. 25788487 10.1098/rsbl.2015.0058 PMC4387502

[evan21515-bib-0064] Kaplan, H. , & Gurven, M. (2008). Top‐down and bottom‐up research in biodemography. Demographic Research, 19, 1587.

[evan21515-bib-0065] Kaplan, H. , Gurven, M. , & Winking, J. (2009). An evolutionary theory of human life span: Embodied capital and the human adaptive complex. Handbook of theories of aging, 39‐60.

[evan21515-bib-0066] Kaplan, H. , Gurven, M. , Winking, J. , Hooper, P. L. , & Stieglitz, J. (2010). Learning, menopause, and the human adaptive complex. Annals New York Academy of Sciences, 1204, 30–42. 10.1111/j.1749-6632.2010.05528.x PMC13184897 20738273

[evan21515-bib-0067] Kaplan, H. , Hill, K. , Lancaster, J. B. , & Hurtado, A. M. (2000). A theory of human life history evolution: Diet, intelligence, and longevity. Evolutionary Anthropology, 9, 156–185.

[evan21515-bib-0068] Kaplan, H. , Hooper, P. L. , Stieglitz, J. , & Gurven, M. (2015). The causal relationship between fertility and infant mortality. *Population in the Human Sciences: Concepts, Models* , Evidence, 361–376

[evan21515-bib-0069] Kaplan, H. S. (1997). The evolution of the human life course In Wachter K. & Finch C. (Eds.), Between Zeus and Salmon: the biodemography of aging (pp. 175‐211). Washington, D.C: National Academy of Sciences.

[evan21515-bib-0070] Kaplan, H. S. , Hooper, P. L. , & Gurven, M. (2009). The evolutionary and ecological roots of human social organization. Philosophical Transactions of the Royal Society B: Biological Sciences, 364(1533), 3289–3299. 10.1098/rstb.2009.0115 PMC2781874 19805435

[evan21515-bib-0071] Kaplan, H. S. , & Robson, A. J. (2002). The emergence of humans: the coevolution of intelligence and longevity with intergenerational transfers. Proceedings of the National Academy of Sciences, 99, 10221–10226. 10.1073/pnas.152502899 PMC126651 12122210

[evan21515-bib-0072] Kirkwood, T. B. L. (1990). The disposable soma theory of aging In Harrison D. E. (Ed.), Genetic Effects on Aging II (pp. 9–19). Caldwell, N.J: Telford Press.

[evan21515-bib-0073] Lee, R. B. , & DeVore, I. (1976). Kalahari hunter‐gatherers: Studies of the! Kung San and their neighbors: Harvard Univ Pr.

[evan21515-bib-0074] Lee, R. D. (2008). Sociality, selection and survival: simulated evolution of mortality with intergenerational transfers and food sharing. Proceedings of the National Association for Science, USA 105, 7124–7128. 10.1073/pnas.0710234105 PMC2438215 18458325

[evan21515-bib-0075] Marlowe, F. (2010). The Hadza: hunter‐gatherers of Tanzania (Vol.3) Berkeley: University of California Press.

[evan21515-bib-0076] Marlowe, F. W. , Berbesque, J. C. , Barr, A. , Barrett, C. , Bolyanatz, A. , Cardenas, J. C. , … Henrich, J. (2008). More ‘altruistic'punishment in larger societies. Proceedings of the Royal Society of London B: Biological Sciences, 275(1634), 587–592. 10.1098/rspb.2007.1517 PMC2596817 18089534

[evan21515-bib-0077] Marlowe, F. W. , Berbesque, J. C. , Barrett, C. , Bolyanatz, A. , Gurven, M. , & Tracer, D. (2010). The ‘spiteful'origins of human cooperation. *Proceedings of the Royal Society of London B* : Biological Sciences, rspb20102342. 10.1098/rspb.2010.2342 PMC3107632 21159680

[evan21515-bib-0078] Martin, M. , Blackwell, A. , Gurven, M. , & Kaplan, H. (2013). Make New Friends and Keep the Old? Parasite Coinfection and Comorbidity in Homo sapiens In Brinkworth J. F. & Pechenkina K. (Eds.), Primates, Pathogens, and Evolution (pp. 363–387): Springer New York.

[evan21515-bib-0079] Martin, M. , Garcia, G. , Kaplan, H. , & Gurven, M. (2016). Conflict or congruence? Maternal and infant‐centric factors associated with shorter exclusive breastfeeding durations among the Tsimane. Social Science & Medicine, 170, 9–16. 27732906 10.1016/j.socscimed.2016.10.003 PMC5107317

[evan21515-bib-0080] Martin, M. A. , Lassek, W. D. , Gaulin, S. J. , Evans, R. W. , Woo, J. G. , Geraghty, S. R. , Davidson, B.S. , Morrow, A.L. , Kaplan, H.S. , & Gurven, M. D. (2012). Fatty acid composition in the mature milk of Bolivian forager‐horticulturalists: controlled comparisons with a US sample. Maternal & child nutrition, 8(3), 404–418. 22624983 10.1111/j.1740-8709.2012.00412.x PMC3851016

[evan21515-bib-0081] McAllister, L. , Gurven, M. , Kaplan, H. , & Stieglitz, J. (2012). Why do women have more children than they want? Understanding differences in women's ideal and actual family size in a natural fertility population. American Journal of Human Biology, 24, 786–799. doi:10.1002/ajhb.22316 22987773 10.1002/ajhb.22316 PMC3806294

[evan21515-bib-0082] Miner, E. J. , Gurven, M. , Kaplan, H. , & Gaulin, S. J. (2014). Sex difference in travel is concentrated in adolescence and tracks reproductive interests. Proceedings of the Royal Society of London B: Biological Sciences, 281(1796), 20141476. 10.1098/rspb.2014.1476 PMC4213637 25320169

[evan21515-bib-0083] Pisor, A. C. , & Gurven, M. (2016). Risk buffering and resource access shape valuation of out‐group strangers. Scientific Reports, 6, 30435. 27470126 10.1038/srep30435 PMC4965756

[evan21515-bib-0084] Pisor, A. C. , Gurven, M. , Blackwell, A. D. , Kaplan, H. , & Yetish, G. (2013). Patterns of senescence in human cardiovascular fitness: VO2max in subsistence and industrialized populations. American Journal of Human Biology, 25(6), 756–769. 24022886 10.1002/ajhb.22445 PMC4142762

[evan21515-bib-0085] Rook, G. A. (2012). Hygiene hypothesis and autoimmune diseases. Clinical reviews in allergy & immunology, 42, 5–15. 22090147 10.1007/s12016-011-8285-8

[evan21515-bib-0086] Rucas, S. , Gurven, M. , Kaplan, H. , Winking, J. , Gangestad, S. , & Crespo, M. (2006). Female intrasexual competition and reputational effects on attractiveness among the Tsimane of Bolivia. Evolution and Human Behavior, 27, 40–52.

[evan21515-bib-0087] Rucas, S. , Gurven, M. , Winking, J. , & Kaplan, H. (2012). Social aggression and resource conflict across the female life‐course in the Bolivian Amazon. Aggressive Behavior, 38, 194–207. 22531995 10.1002/ab.21420

[evan21515-bib-0088] Rucas, S. L. , Gurven, M. , Kaplan, H. , & Winking, J. (2010). The Social Strategy Game. Human Nature, 21(1), 1–18. 20526460 10.1007/s12110-010-9079-z PMC2879671

[evan21515-bib-0089] Schniter, E. , Gurven, M. , Kaplan, H. S. , Wilcox, N. T. , & Hooper, P. L. (2015). Skill ontogeny among Tsimane forager‐horticulturalists. American Journal of Physical Anthropology, 158(1), 3–18. 25921880 10.1002/ajpa.22757

[evan21515-bib-0090] Sell, A. , Bryant, G. A. , Cosmides, L. , Tooby, J. , Sznycer, D. , Von Rueden, C. , Krauss, A. , & Gurven, M. (2010). Adaptations in humans for assessing physical strength from the voice. Proceedings of the Royal Society of London B: Biological Sciences, 277(1699), 3509–3518. 10.1098/rspb.2010.0769 PMC2982226 20554544

[evan21515-bib-0091] Sell, A. , Cosmides, L. , Tooby, J. , Sznycer, D. , von Rueden, C. , & Gurven, M. (2009). Human adaptations for the visual assessment of strength and fighting ability from the body and face. *Proceedings of the Royal Society of London* B: Biological Sciences, 276(1656), 575–584. 10.1098/rspb.2008.1177 PMC2664345 18945661

[evan21515-bib-0092] Stearns, S. C. (1992). The Evolution of Life Histories. Oxford: Oxford University Press.

[evan21515-bib-0093] Stieglitz, J. , Beheim, B. A. , Trumble, B. C. , Madimenos, F. C. , Kaplan, H. , & Gurven, M. (2015). Low mineral density of a weight‐bearing bone among adult women in a high fertility population. American Journal of Physical Anthropology, 156, 637–648. 25488367 10.1002/ajpa.22681 PMC4368479

[evan21515-bib-0094] Stieglitz, J. , Blackwell, A. D. , Gutierrez, R. Q. , Linares, E. C. , Gurven, M. , & Kaplan, H. (2012). Modernization, sexual risk‐taking, and gynecological morbidity among Bolivian forager‐horticulturalists. PLoS ONE, 7, e50384. 23236371 10.1371/journal.pone.0050384 PMC3516519

[evan21515-bib-0095] Stieglitz, J. , Gurven, M. , Kaplan, H. , & Hooper, P. (2013). Household Task Delegation among High‐Fertility Forager‐Horticulturalists of Lowland Bolivia. Current Anthropology, 54(2), 232–241. 25284827 10.1086/669708 PMC4184416

[evan21515-bib-0096] Stieglitz, J. , Gurven, M. , Kaplan, H. , & Hopfensitz, A. (2016). Why household inefficiency? An experimental approach to assess spousal resource distribution preferences in a subsistence population undergoing socioeconomic change. Evolution and Human Behavior.

[evan21515-bib-0097] Stieglitz, J. , Gurven, M. , Kaplan, H. , & Winking, J. (2012). Infidelity, jealousy, and wife abuse among Tsimane forager–farmers: testing evolutionary hypotheses of marital conflict. Evolution and Human Behavior, 33, 438–448. 23459748 10.1016/j.evolhumbehav.2011.12.006 PMC3583221

[evan21515-bib-0098] Stieglitz, J. , Jaeggi, A. , Blackwell, A. , Trumble, B. , Gurven, M. , & Kaplan, H. (2014). Work to live and live to work: Productivity, transfers, and psychological well‐being in adulthood and old age. In M. Weinstein & M. Lane (Eds.), *Sociality, Hierarchy, and Health: comparative biodemography* (pp. 197‐221). Washington DC: Committee on Population of the National Research Council, National Academies Press.

[evan21515-bib-0099] Stieglitz, J. , Kaplan, H. , Gurven, M. , Winking, J. , & Tayo, B. V. (2011). Spousal violence and paternal disinvestment among Tsimane forager‐horticulturalists. American Journal of Human Biology, 23, 445–457. 21547978 10.1002/ajhb.21149

[evan21515-bib-0100] Stieglitz, J. , Madimenos, F. , Kaplan, H. , & Gurven, M. (2016). Calcaneal Quantitative Ultrasound Indicates Reduced Bone Status Among Physically Active Adult Forager‐Horticulturalists. Journal of Bone and Mineral Research, 31(3), 663–671. 26460548 10.1002/jbmr.2730 PMC4834389

[evan21515-bib-0101] Stieglitz, J. , Schniter, E. , Von Rueden, C. , Kaplan, H. , & Gurven, M. (2014). Functional disability and social conflict increase risk of depression in older adulthood among Bolivian forager‐farmers. *The Journals of Gerontology Series B: Psychological Sciences and Social Sciences*, gbu080. 10.1093/geronb/gbu080 PMC4841159 24986182

[evan21515-bib-0102] Stieglitz, J. , Trumble, B. C. , Thompson, M. E. , Blackwell, A. D. , Kaplan, H. , & Gurven, M. (2015). Depression as sickness behavior? A test of the host defense hypothesis in a high pathogen population. Brain, Behavior, and Immunity, 49, 130–139. 10.1016/j.bbi.2015.05.008 PMC4567437 26044086

[evan21515-bib-0103] Straub, R. H. , Cutolo, M. , Buttgereit, F. , & Pongratz, G. (2010). Energy regulation and neuroendocrine–immune control in chronic inflammatory diseases. Journal of Internal Medicine, 267, 543–560. 20210843 10.1111/j.1365-2796.2010.02218.x

[evan21515-bib-0104] Thompson, R. C. , Allam, A. H. , Lombardi, G. P. , Wann, L. S. , Sutherland, M. L. , Sutherland, J. D. , Al‐Tohamy Soliman, M. , Frohlich, B. , Mininberg, D.T. , Monge, J.M. , Vallodolid, C.M. , Cox, S.M. , Abd el‐Maksoud, G. , Badr, I. , Miyamoto, M.I. , el‐Halim Nur el‐din, A. , Narula, J. , Finch, C.E. , & Thomas, G.S. (2013). Atherosclerosis across 4000 years of human history: the Horus study of four ancient populations. The Lancet, 381, 1211–1222. 10.1016/S0140-6736(13)60598-X 23489753

[evan21515-bib-0105] Trumble, B. C. , Blackwell, A. D. , Stieglitz, J. , Thompson, M. E. , Suarez, I. M. , Kaplan, H. , & Gurven, M. (2016). Associations between male testosterone and immune function in a pathogenically stressed forager‐horticultural population. *American Journal of Physical Anthropology*. 10.1002/ajpa.23054 PMC5075254 27465811

[evan21515-bib-0106] Trumble, B. C. , Cummings, D. , von Rueden, C. , O'Connor, K. A. , Smith, E. A. , Gurven, M. , & Kaplan, H. (2012). *Physical competition increases testosterone among Amazonian forager‐horticulturalists: a test of the ‘challenge hypothesis’*. Paper presented at the Proc. R. Soc. B. 10.1098/rspb.2012.0455 PMC3367794 22456888

[evan21515-bib-0107] Trumble, B. C. , Cummings, D. K. , O'Connor, K. A. , Holman, D. J. , Smith, E. A. , Kaplan, H. S. , & Gurven, M. D. (2013). Age‐independent increases in male salivary testosterone during horticultural activity among Tsimane forager‐farmers. Evolution and Human Behavior, 34(5), 350–357. 10.1016/j.evolhumbehav.2013.06.002 PMC3810999 24187482

[evan21515-bib-0108] Trumble, B. C. , Gaulin, S. J. , Dunbar, M. D. , Kaplan, H. , & Gurven, M. (2016). No sex or age difference in dead‐reckoning ability among Tsimane forager‐horticulturalists. Human Nature, 27(1), 51–67. 26590826 10.1007/s12110-015-9246-3

[evan21515-bib-0109] Trumble, B. C. , Smith, E. A. , O'Connor, K. A. , Kaplan, H. S. , & Gurven, M. D. (2014). Successful hunting increases testosterone and cortisol in a subsistence population. Proceedings of the Royal Society of London B: Biological Sciences, 281(1776). doi:10.1098/rspb.2013.2876 10.1098/rspb.2013.2876 PMC3871326 24335989

[evan21515-bib-0110] Trumble, B. C. , Stieglitz, J. , Rodriguez, D. E. , Linares, E. C. , Kaplan, H. S. , & Gurven, M. D. (2015). Challenging the Inevitability of prostate enlargement: Low levels of benign prostatic hyperplasia among Tsimane forager‐horticulturalists. The Journals of Gerontology Series A: Biological Sciences and Medical Sciences, 70(10), 1262–1268. 10.1093/gerona/glv051 PMC4851713 25922348

[evan21515-bib-0111] Trumble, B. C. , Stieglitz, J. , Thompson, M. E. , Fuerstenberg, E. , Kaplan, H. , & Gurven, M. (2015). Testosterone and male cognitive performance in Tsimane forager‐horticulturalists. American Journal of Human Biology, 27(4), 582–586. doi:10.1002/ajhb.22665 25429990 10.1002/ajhb.22665 PMC8446946

[evan21515-bib-0112] Tuljapurkar, S. D. , Puleston, C. O. , & Gurven, M. D. (2007). Why men matter: mating patterns drive evolution of human lifespan. PLoS ONE, 2(8), e785. 17726515 10.1371/journal.pone.0000785 PMC1949148

[evan21515-bib-0113] Vasunilashorn, S. , Crimmins, E. M. , Kim, J. K. , Winking, J. , Gurven, M. , Kaplan, H. , & Finch, C. E. (2010). Blood lipids, infection, and inflammatory markers in the Tsimane of Bolivia. American Journal of Human Biology, 22(6), 731–740. doi:10.1002/ajhb.21074 20721985 10.1002/ajhb.21074 PMC3537506

[evan21515-bib-0114] Vasunilashorn, S. , Finch, C. E. , Frimmins, E. M. , Vikman, S. A. , Stieglitz, J. , Gurven, M. , Kaplan, H. , & Allayee, H. (2011). Inflammatory gene variants in the Tsimane, an indigenous Bolivian population with a high infectious load. Biodemography and Social Biology, 57(1), 33–52. 21845926 10.1080/19485565.2011.564475 PMC3529658

[evan21515-bib-0115] Veile, A. , Martin, M. A. , McAllister, L. , & Gurven, M. (2014). Modernization and traditional breastfeeding patterns in the Bolivian Amazon. Social Science & Medicine, 100, 148–158. 24444850 10.1016/j.socscimed.2013.10.034 PMC4093802

[evan21515-bib-0116] Veile, A. , Winking, J. , Gurven, M. , Greaves, R. D. , & Kramer, K. L. (2012). Infant growth and the thymus: data from two South American native societies. *American* Journal of Human Biology, 24(6), 768–775. 10.1002/ajhb.22314 22915311

[evan21515-bib-0117] von Rueden, C. , Gurven, M. , & Kaplan, H. (2008). The multiple dimensions of male social status in an Amazonian society. Evolution and Human Behavior, 29(6), 402–415. 19884954 10.1016/j.evolhumbehav.2008.05.001 PMC2598750

[evan21515-bib-0118] von Rueden, C. , Gurven, M. , & Kaplan, H. (2011). Why do men seek status? Fitness payoffs to dominance and prestige. *Proceedings of the Royal Society* B: Biological Sciences, 278, 2223–2232. 10.1098/rspb.2010.2145 PMC3107626 21147798

[evan21515-bib-0119] von Rueden, C. , Gurven, M. , Kaplan, H. , & Stieglitz, J. (2014). Leadership in an egalitarian society. Human Nature, 25(4), 538–566. 25240393 10.1007/s12110-014-9213-4 PMC4258461

[evan21515-bib-0120] von Rueden, C. R. , Lukaszewski, A. W. , & Gurven, M. (2015). Adaptive personality calibration in a human society: effects of embodied capital on prosocial traits. Behavioral Ecology, 26, 1071–1082.

[evan21515-bib-0121] von Rueden, C. R. , Trumble, B. C. , Thompson, M. E. , Stieglitz, J. , Hooper, P. L. , Blackwell, A. D. , Kaplan, H. , & Gurven, M. (2014). Political influence associates with cortisol and health among egalitarian forager‐farmers. Evolution, medicine, and public health, 2014, 122–133. 10.1093/emph/eou021 PMC4178369 25214482

[evan21515-bib-0122] Walker, R. , Gurven, M. , Hill, K. I. M. , Migliano, A. , Chagnon, N. , De Souza, R. , Djurovic, G. , Hames, R. , Hurtado, A.M. , Kaplan, H. , Kramer, K. , Oliver, W.J. , Valeggia, C. , & Yamauchi, T. (2006). Growth Rates and Life Histories in Twenty‐Two Small‐Scale Societies. American Journal of Human Biology, 18, 295–311. 16634027 10.1002/ajhb.20510

[evan21515-bib-0123] Walker, R. , Hill, K. , Kaplan, H. , & McMillan, G. (2002). Age‐dependency in skill, strength and hunting ability among the Ache of eastern Paraguay. Journal of Human Evolution, 42, 639–657. 12069505 10.1006/jhev.2001.0541

[evan21515-bib-0124] Walker, R. S. , Beckerman, S. , Flinn, M. V. , Gurven, M. , von Rueden, C. R. , Kramer, K. L. , Hill, K. , Gurven, M. , von Rueden, C. , Beckerman, S. , Greaves, R.D. , Cordoba, L. , Villar, D. , Hagen, E.H. , Koster, J.M. , Sugiyama, L. , & Hunter, T.E. (2013). Living with kin in lowland horticultural societies. Current Anthropology, 54(1), 96–103.

[evan21515-bib-0125] Walker, R. S. , Gurven, M. , Burger, O. , & Hamilton, M. J. (2008). The trade‐off between number and size of offspring in humans and other primates. Proceedings of the Royal Society of London B: Biological Sciences, 275(1636), 827–834. 10.1098/rspb.2007.1511 PMC2596903 18077252

[evan21515-bib-0126] Weiss, A. , King, J. E. , Inoue‐Murayama, M. , Matsuzawa, T. , & Oswald, A. J. (2012). Evidence for a midlife crisis in great apes consistent with the U‐shape in human well‐being. Proceedings of the National Academy of Sciences, 109, 19949–19952. 10.1073/pnas.1212592109 PMC3523857 23169637

[evan21515-bib-0127] Winking, J. (2006). Are men really that bad as fathers? The role of men's investments. Social Biology, 53(1‐2), 100–115. 21516953 10.1080/19485565.2006.9989119

[evan21515-bib-0128] Winking, J. , & Gurven, M. (2011). The total cost of father desertion. American Journal of Human Biology, 23(6), 755–763. 21932418 10.1002/ajhb.21207

[evan21515-bib-0129] Winking, J. , Gurven, M. , & Kaplan, H. (2011a). Father death and adult success among the Tsimane: implications for marriage and divorce. Evolution and Human Behavior, 32(2), 79–89. 21516217 10.1016/j.evolhumbehav.2010.08.002 PMC3079911

[evan21515-bib-0130] Winking, J. , Gurven, M. , & Kaplan, H. (2011b). The impact of parents and self‐selection on child survival among the Tsimane of Bolivia. Current Anthropology, 52(2), 277–284.

[evan21515-bib-0131] Winking, J. , Gurven, M. , Kaplan, H. , & Stieglitz, J. (2009). The goals of direct paternal care among a South Amerindian population. American Journal of Physical Anthropology, 139, 295–304. 19140194 10.1002/ajpa.20981

[evan21515-bib-0132] Winking, J. , Kaplan, H. , Gurven, M. , & Rucas, S. (2007). Why do men marry and why do they stray? *Proceedings of the Royal Society* Biological Sciences, 274, 1643–1649. 17456459 10.1098/rspb.2006.0437 PMC2169272

[evan21515-bib-0133] Winking, J. , Stieglitz, J. , Kurten, J. , Kaplan, H. , & Gurven, M. (2013). Polygyny among the Tsimane of Bolivia: an improved method for testing the polygyny‐fertility hypothesis. Proceedings of the Royal Society B: Biological Sciences, 280, 20123078. 23407840 10.1098/rspb.2012.3078 PMC3574385

[evan21515-bib-0134] Wiria, A. E. , Sartono, E. , Supali, T. , & Yazdanbakhsh, M. (2014). Helminth infections, type‐2 immune response, and metabolic syndrome. PLoS Pathogens, 10, doi:10.1371/journal.ppat.1004140. 10.1371/journal.ppat.1004140 PMC4081794 24992724

[evan21515-bib-0135] Yazdanbakhsh, M. , Kremsner, P. G. , & van Ree, R. (2002). Allergy, parasites, and the hygiene hypothesis. Science, 296, 490–494. 11964470 10.1126/science.296.5567.490

[evan21515-bib-0136] Yetish, G. , Kaplan, H. , Gurven, M. , Wood, B. , Pontzer, H. , Manger, P. R. , Wilson, C. , McGregor, R. , & Siegel, J. M. (2015). Natural sleep and its seasonal variations in three pre‐industrial societies. Current Biology, 25(21), 2862–2868. 26480842 10.1016/j.cub.2015.09.046 PMC4720388

